# A Review of *Aronia melanocarpa*’s Phytochemical Profile, Health Benefits, and Applications in Agri-Food Systems

**DOI:** 10.3390/foods15101627

**Published:** 2026-05-07

**Authors:** Jingchun Chen, Ziyue Hu, Shifeng Chen, Yiling Yao, Xinyue Wang, Wanyi Zou, Xiaoni Shao

**Affiliations:** College of Pharmacy and Food, Southwest Minzu University, Chengdu 610225, China; chen0604062025@126.com (J.C.); 18327589911@163.com (Z.H.); 17304630273@163.com (S.C.); yyl10104825@163.com (Y.Y.); wxy2005915815@163.com (X.W.); wwsunny7725@163.com (W.Z.)

**Keywords:** *Aronia melanocarpa*, polyphenols, anthocyanins, pharmacological mechanism, ferroptosis, functional food, antioxidant, agri-food applications

## Abstract

*Aronia melanocarpa* (commonly known as black chokeberry) is a Rosaceae species native to eastern North America that has long been recognized as both a food and medicinal plant. Its berries are rich in polyphenols, particularly anthocyanins such as cyanidin-3-O-galactoside (19–1282 mg/100 g), which are associated with a wide range of bioactivities, including antioxidant, anti-inflammatory, hepatoprotective, neuroprotective, cardiometabolic-regulating, and antitumor effects. These activities involve key signaling pathways such as Nrf2/KEAP1, NF-κB/MAPK, and PI3K/Akt, as well as inhibition of ferroptosis. The rich phytochemical profile of aronia supports diverse applications in the agri-food sector, including sports nutrition products, natural antioxidant additives, natural pigments, food preservation, and food coloring. Based on a systematic search of Scopus, PubMed, Web of Science, Google Scholar, Taylor & Francis, ScienceDirect, CNKI, and Wanfang Data up to October 2025, this narrative review summarizes the latest advances in aronia cultivation, chemical composition, pharmacological mechanisms, and food and clinical applications. Despite the growing body of evidence, significant gaps remain: clinical studies in humans are still limited, standardized extracts are lacking, and little is known about how these bioactive compounds behave during food processing and storage. This review highlights these gaps and outlines future research directions to maximize the potential of aronia for promoting human health.

## 1. Introduction

*Aronia melanocarpa* (aronia) is an edible shrub belonging to the family Rosaceae native to the eastern region of North America [1]. Due to its reputation as a highly nutritious and health-promoting plant, it has attracted considerable global attention and is now widely cultivated for both culinary and medicinal purposes [1,2].

The large-scale cultivation of aronia supplies the raw materials needed for its use in food products. A growing number of people are now considering the complete supply chain of functional foods, from agricultural production through to consumer consumption [3,4]. The nutritional profile of berries is heavily influenced by both varietal differences and growing conditions [5,6,7]. Notably, aronia is distinguished by its higher amino acid content compared to blueberries. Furthermore, a study on South Korean aronia demonstrated that amino acid concentrations, along with metabolites such as sucrose, can vary significantly depending on the specific cultivar and the latitude of cultivation [8]. The nutrient content of a berry largely governs its suitability for various food products and its overall commercial value. This nutritional profile, in turn, provides the foundation for the health-related characteristics attributed to aronia [1].

Within the food industry, aronia is widely used in the development of diversified products, such as juices, yogurts, jams, syrups and tea beverages [9,10,11,12,13,14]. In addition, aronia can also be used as an athletic nutrition supplement, which may improve various aspects of an athlete’s health and ultimately enhance their performance [12,15,16,17,18]. Beyond its basic nutritional value, the strong antioxidant properties of aronia allow it to be utilized as a multifunctional food additive. For instance, as a natural pigment, an antioxidant agent, or an edible preservation coating. It can thus serve as a healthy, non-toxic alternative to synthetic preservatives such as sodium nitrite [18,19,20,21,22,23]. These applications not only meet the growing demand of consumers for functional foods but also further enhance the commercial value of aronia.

These industrial applications depend on the unique chemical composition and functional properties of aronia. Recent studies have explored its active components [24]. It has been identified that aronia is rich in phenolic and terpenoid compounds [25,26,27]. Small amounts of lipids, organic acids, vitamins, and sterols are also present [2,24,27,28,29]. These bioactive substances explain certain biological effects and are thus responsible for aronia’s excellent health-promoting properties [30].

Various compounds have been isolated and identified in aronia. Polyphenolic compounds stand out for their excellent antioxidant capacity and constitute the main material basis for the antioxidant activity of aronia. Especially anthocyanins are a powerful source of natural antioxidants [31]. Studies have shown aronia’s antioxidant effects by regulating the Nrf2/Keap1 pathway. This antioxidant effect gives aronia broad anti-inflammatory effects. It lowers reactive oxygen species. It inhibits NF-κB nuclear translocation and down-regulates the expression of pro-inflammatory mediators like TNF-α, IL-6, and COX-2 [31,32]. Oxidative stress and inflammation drive chronic diseases [33]. Studies have shown that the extract of aronia fruits and its bio-active substances possess hepatoprotective effects [1,34,35] anti-tumor effects [36,37,38,39], neuroprotective effects [40], and anti-diabetic and anti-obesity effects [41].

Several previous reviews have focused on specific aspects of aronia, such as its phytochemical composition, its effects on particular diseases, or its processing techniques [1,2,24,30,34,42,43,44,45]. However, a comprehensive synthesis that integrates phytochemical profiles, health benefits, and food applications within the context of modern agri-food systems has been lacking. This review aims to fill this gap by providing a systematic overview of aronia from cultivation to functional food development. Specifically, this review categorized the phytochemical constituents of aronia with detailed information on concentrations and extraction methods, and evidence-based connections between major bioactive compounds and their pharmacological mechanisms are established. In addition, this review proposes a novel mechanistic link be-tween quercetin-rich aronia extracts and ferroptosis, illustrated through a comprehensive pathway diagram.

## 2. Methodology

A systematic literature search was performed across multiple international databases, including Scopus, PubMed, Web of Science, Google Scholar, Taylor & Francis, Science Direct, CNKI, and Wanfang Data. The search covered publications from the inception of each database to February 2026. The search utilized specific keywords, including: *Aronia melanocarpa*, black chokeberry, polyphenols, anthocyanins, flavonoids, antioxidant, anti-inflammatory, hepatoprotective, neuroprotective, antidiabetic, anti-obesity, anti-fibrotic, Nrf2, NF-κB, PI3K/Akt, MAPK, ferroptosis, functional food, food additive, food application, and clinical trial. Boolean operators (AND, OR) were used to combine search terms as appropriate. Given the iterative nature of the review process, the literature search was continuously updated throughout manuscript preparation to ensure inclusion of the most recent publications. Studies were included if they were original research or peer-reviewed reviews on the chemical components, pharmacological mechanisms, or food applications of *Aronia melanocarpa*. Master’s and doctoral theses containing original experimental data not available in other published sources were also considered when they provided essential information for specific aspects of this review. Non-peer-reviewed abstracts, conference proceedings, and studies without full-text access were excluded. Two authors independently screened the titles and abstracts of the retrieved articles based on relevance to the scope of this review. The final selection included 162 peer-reviewed articles and relevant theses, covering aspects of cultivation, chemical composition, pharmacological mechanisms, food applications, and clinical studies.

## 3. Agronomic Practices and Sustainability in Agri-Food Systems

### 3.1. Origin and Global Dissemination

Aronia is native to the wetlands and forest edges of eastern North America. From its center of origin, it was later introduced into Eastern Europe (e.g., Russia and Poland) at the beginning of the 20th century due to its hardiness and nutritious fruit [46]. Later it was recognized also for its beneficial properties on human health [34,47] and has been introduced and cultivated commercially in the temperate world, e.g., in Scandinavia, Central Europe, and some areas in Asia [48]. Its great adaptability in the field, including poor drainage and acid soils, along with pest and disease tolerance or resistance, has also played an important role in its successful establishment and cultivation as a low-input crop. From an agri-food systems perspective, this resilience makes aronia well-suited for sustainable agriculture, including on land that is too poor or difficult for traditional fruit farming [49].

### 3.2. Major Commercial Cultivars

Through selective breeding programs, several improved cultivars have been developed [50], primarily focusing on enhancing yield, fruit size, anthocyanin content, and agronomic traits. The most widely cultivated and commercially significant genotypes include.

1. “Viking”: The most common cultivar globally. It has a vigorous growth habit, high productivity, and produces medium-sized berries with excellent black coloration and high polyphenol content. Primary use: juice processing and functional food ingredients.
2. “Nero”: Widely cultivated in Poland. It is characterized by the highest levels of anthocyanins and total polyphenols, making it ideal for nutraceutical extraction. Berry size is medium to large, with high yield potential. Its high yield and vigorous vigor contribute to its economic viability. Primary use: functional food products.
3. “Hugin”: A Swedish variety with upright growth habit that facilitates mechanical harvesting. Berries are small but have an acceptable sugar-acid balance, making them suitable for juice production. Primary use: juice and juice concentrates.
4. “Aron” (“McKenzie”): A North American selection valued for its cold hardiness and ornamental appeal, alongside consistent fruit production. Berry size is medium, with moderate polyphenol content. Primary use: landscape ornamental with secondary fruit production.
5. “Galicjanka”: A Polish cultivar known for its large fruit size and high juice yield. It is preferred for direct eating and juice processing. Primary use: fresh consumption and premium juice production.

The choice of cultivar depends on its use (e.g., juice production, fresh consumption, or nutraceutical extraction) and local climatic conditions, highlighting the importance of selecting the right variety for optimizing harvest outcomes.

### 3.3. Agronomic Practices, Quality Influencers and Sustainability

Successful cultivation of aronia for optimal bioactive compound production requires attention to several agronomic factors. When managed well, these practices can boost fruit quality while reducing environmental impact:

1. Planting Location: It prefers full sunlight to maximize photosynthesis [51] and anthocyanin synthesis. It tolerates a variety of soils but prefers well-drained, loamy soil with a slightly acidic pH (5.0–6.5).
2. Planting and Spacing: Usually propagated from hardwood cuttings, also by tissue culture. Plantations are usually planted at a distance of 0.8–1.2 m within the row, which is spaced at 3–4 m from adjacent rows, allowing space for machinery passage and good light penetration.
3. Nutrition and Water: Balanced fertilization, especially the avoidance of excess nitrogen, which can cause vegetative growth to be promoted while fruit development and phytochemical accumulation are reduced at the same time; consistent moisture, especially at the time of fruit set and development, is essential to berry quality [52].
4. Harvesting: Fruits are fully mature and have maximum levels of anthocyanins between August and September (Northern Hemisphere) when harvesting occurs, usually by mechanical means, after they attain a dark black-purple color and their soluble solids content reaches desirable values.
5. Harvesting and Post-Harvest Handling: Berries have a very high polyphenol oxidase activity so they can easily suffer from enzymatic browning; rapid cooling, drying (if the product is to be dried), or quick processing (if the product is to be made into a juice/concentrate) after harvest are critical to maintaining bioactive intactness as well as color [53].

The way berries are handled after harvest directly affects the quality of final food products—connecting the farm to the fork [54].

### 3.4. Influence of Genotype and Environment on Phytochemical Profile

The content of bioactive compounds shows high variability among cultivars and within cultivars due to their genetic background and cultivation conditions (genotype × environment effect). In most cases, “Nero” has been reported to contain a significantly greater total amount of anthocyanins than “Viking” grown in the same environment. Additionally, mild water deficit or low night temperature at ripening are abiotic stresses that induce increased biosynthesis of secondary compounds (e.g., anthocyanins/proanthocyanidins) in a protection response, potentially improving the functional quality of the berries in terms of nutraceuticals as well. Therefore, the selection of suitable cultivars based on local climatic conditions and target end-use (e.g., high-contrast vs. high-yield) is an important factor for producers and processors interested in maximizing the functional food value of aronia products [55,56].

### 3.5. By-Products Valorization: Bridging Agriculture and Food Industries

A key part of aronia’s value in agri-food systems is the conversion of agricultural by-products (pomace) into useful resources [57]. Juice processing generates substantial quantities of pomace (seeds, skins, and residual pulp), which was once regarded as waste. However, this by-product is increasingly recognized as a valuable resource for several reasons:

1. Rich Bioactive Compounds: Even after the berries are squeezed for juice, the leftover pomace still holds plenty of valuable compounds—like anthocyanins, fiber, and other polyphenols [25]. Quantitative analysis shows that anthocyanin content in pomace can reach 0.5–1120 mg/100 g dry weight, which is 1.5 to 2.5 times higher than in juice (13–787 mg/100 g dry weight), which makes it an excellent source for making natural food dyes [22,58].
2. Circular Economy Potential: Instead of being thrown away, pomace can be processed into valuable ingredients for the food industry. For example, dried pomace powder can be used as a natural food coloring [22,59], a fiber supplement, or a functional food ingredient [10,60].
3. Sustainability Implications: Using pomace reduces farm waste, brings extra income to growers and processors [61,62], and lessens the environmental impact of aronia production [63,64]. This represents a clear example of circular economy thinking—where leftovers from one industry become valuable resources for another [65].
4. Emerging Applications: Recent studies have explored new ways to use aronia pomace extracts—for example, in active food packaging [23,66], as natural preservatives [19,61], and even in animal feed to support livestock health [62].

Turning pomace into valuable products not only generates additional income but also supports the global shift toward sustainable food production and waste reduction. These are at the heart of modern agri-food systems.

## 4. Chemical Composition

Aronia contains many different bioactive compounds. These include phenolic compounds, terpenoids, lipids, organic acids, vitamins, sterols, and other substances. They collectively contribute to its multiple physiological activities and health benefits. To systematically summarize the chemical composition of aronia, compounds are listed in Table 1, which provides detailed information on the chemical names, molecular formulas, content (where available), and analytical methods used for their identification and quantification. This table serves as a comprehensive reference for the phytochemical characterization of this plant material.

### 4.1. Phenolic Compounds

Polyphenols are a class of natural organic compounds widely present in plants, exhibiting antioxidant and anti-inflammatory properties [67]. *Aronia melanocarpa* also contains significant quantities of polyphenolic compounds. *Aronia melanocarpa* is recognized as the richest source of polyphenols among various berries, being abundant in proanthocyanidins, anthocyanins and phenolic acids, while exhibiting lower levels of flavanones and flavanonols [25]. These polyphenols have been shown to act synergistically to combat diseases and inhibit viral enzymes. For example, quercetin and ellagic acid, along with other phytochemicals, have demonstrated potential anti-viral activity against SARS-CoV-2 when they bind to viral proteins. Concurrently, the polyphenolic compounds within the fruit can mitigate oxidative damage induced by oxidants. Anthocyanins were extracted from leaves and subjected to antioxidant activity testing. The results revealed a consistent trend between anthocyanin scavenging capacity for DPPH· and OH· radicals and their total reducing power.

**Table 1:** Phytochemical constituents identified and quantified in *A. melanocarpa*.

Class	No.	Name	Molecular Weight	References
Flavonols and Flavanols	1.	Cyanidin-3-O-glucoside	468.84	[25,26,27]
	2.	Cyanidin-3-O-xyloside	386.36	[25]
	3.	Cyanidin-3-O-galactoside	466.40	[25,26,27]
	4.	Cyanidin-3-O-β-galactoside	466.40	[28]
	5.	Hyperin	464.38	[25]
	6.	Quercetin-3-O-rutinoside	610.52	[25]
	7.	Quercetin-3-O-rhamnoside	448.38	[25,68]
	8.	Quercetin-3-O-arabinoside	434.35	[29]
	9.	(−)-Epicatechin	290.27	[25]
	10.	(−)-Gallocatechin	306.27	[25]
	11.	Quercetin	302.24	[69]
	12.	Quercetin-3-O-galactoside	464.38	[27]
	13.	Quercetin-3-O-β-D-galactopyranoside	464.38	[28]
	14.	Quercetin-3-O-β-D-glucopyranoside	464.38	[28]
	15.	Rutin	610.52	[69]
	16.	Cyanidin-3-O-arabinoside	438.81	[25]
	17.	Pelargonidin-3-O-arabinoside	420.37	[25,26]
	18.	Pelargonidin-3-O-galactoside	468.84	[25]
	19.	Quercetin-3-O-glucoside	478.36	[25,68]
	20.	Quercetin-3-O-xyloside	434.35	[25]
	21.	Kaempferol	286.24	[25]
	22.	Myricetin	318.24	[25]
	23.	Eriodictyol-7-O-glucuronide	464.38	[25]
	24.	Cyanidin Chloride	287.24	[25]
	25.	Quercetin 3-O-vicianoside	596.49	[68]
	26.	Quercetin-O-dihexoside	302.23	[24,28,68]
	27.	Quercetin-O-deoxyhexoside-hexoside	594.50	[24]
	28.	Quercetin-diglucoside	626.52	[24]
	29.	Quercetin-sulfate	381.30	[29]
	30.	Myricetin-3-O-glucoside	480.38	[24]
	31.	Myricetin-3-O-galactoside	480.09	[24]
	32.	Isorhamnetin 3-O-rutinoside	624.54	[24,68]
	33.	Isorhamnetin 3-O-neohesperidoside	624.54	[24]
	34.	Eriodictyol-glucuronide	464.38	[24]
Anthocyanins	35.	Eriodictyol-3,7-O-diglucuronide	464.38	[24]
	36.	Naringenin	272.25	[24]
	37.	Phloretin	274.27	[29]
	38.	Resveratrol	228.24	[70]
	39.	Cyanidin	322.70	[71]
	40.	Delphinidin	338.70	[71]
	41.	Malvidin	366.75	[71]
	42.	Peonidin	336.72	[71]
	43.	Pelargonidin	630.98	[71]
	44.	Petunidin	514.86	[71]
	45.	Cyanidin-3,5-O-dihexoside	610.52	[72]
	46.	Cyanidin-3,5-hexoside-(epi)catechin	871.80	[24]
	47.	Cyanidin-3-pentoside-(epi)catechin	677.62	[24]
	48.	Cyanidin 3-O-rhamnoside	433.40	[28]
	49.	Malvidin 3-O-(6-O-p-coumaroyl) glucoside-4-vinylphenol adduct	745.71	[29]
	50.	Methyl-epigallocatechin	320.29	[29]
	51.	Proanthocyanins	594.52	[69]
	52.	Chalcones	208.26	[69]
	53.	Cyanidin-3-O-rutinoside	595.53	[69]
	54.	Delphinidin-3-glucoside	465.39	[69]
	55.	Malvidin-3-glucoside	493.44	[69]
	56.	Kaempferol-3-O-rutinoside	594.52	[24,70]
Phenolic acids	57.	Chlorogenic acid	354.31	[27,28]
	58.	3-O-Caffeoylquinic acid methyl ester	368.34	[28]
	59.	Neochlorogenic acid	354.31	[25,69]
	60.	Protocatechuic acid	154.12	[25,28]
	61.	Rosmarinic acid	360.31	[25]
	62.	Ellagic acid	302.19	[70]
	63.	3,4-Dihydroxyphenylacetic acid	418.41	[25,69]
	64.	Ferulic acid	194.18	[28]
	65.	Quinic acid	192.17	[28]
	66.	2,5-Dihydroxybenzoic acid	154.12	[28]
	67.	Vanillic acid	168.15	[70]
	68.	4-Hydroxybenzoic acid	138.12	[70]
	69.	Syringic acid	198.17	[70]
	70.	3-Hydroxybenzoic acid	138.12	[70]
	71.	p-Coumaric acid	164.16	[70]
	72.	Cinnamic acid	148.16	[70]
	73.	Methoxycinnamic acid	178.18	[70]
	74.	Caffeic acid glucoside	342.30	[24]
	75.	Salicylic acid	138.12	[24]
	76.	Dicaffeoylquinic acid	516.45	[24]
	77.	3-O-p-Coumaroylquinic acid	338.31	[24]
	78.	Caffeic acid	180.16	[70]
	79.	Gallic acid	170.12	[28,70]
Terpenoids	80.	Friedelin	426.73	[73]
	81.	Betulin	442.73	[73]
	82.	Betulinic acid	454.69	[73]
	83.	23-Hydroxybetulinic acid	472.71	[73]
	84.	Betulinic acid 3β-caffeate	604.87	[73]
	85.	23-Hydroxybetulinic acid 3β-caffeate	663.94	[73]
	86.	2α-Hydroxyoleanolic acid	472.71	[73]
	87.	3-acetyl oleanolic acid	498.75	[74]
	88.	Ursolic acid	456.71	[28,73]
	89.	19α-Hydroxyursolic acid	472.71	[28,73]
	90.	Acetylursolic acid	570.84	[73]
	91.	2α,3α-Dihydroxyursolic acid	472.71	[28,73]
	92.	2α,3α,19α-Trihydroxyursolic acid	504.71	[28]
	93.	Oleanolic acid	456.70	[28]
	94.	3β-Hydroxyursane	411.69	[28]
	95.	3β-O-Acetylpomolic acid	514.75	[28]
	96.	(3β)-3,19-Dihydroxy-2-oxours-12-en-28-oic acid	486.69	[28]
	97.	2α,3α,19α-Trihydroxy oleanolic acid	504.37	[28]
	98.	Limonene	136.23	[29]
	99.	Italicene epoxide	220.35	[29]
	100.	Khusinol	220.35	[29,69]
	101.	3,9-Epoxy-p-menth-1-ene	138.25	[27,75]
	102.	3-O-trans-p-coumaroyltormentic acid	634.85	[30]
	103.	3-O-cis-p-coumaroyltormentic acid	634.85	[30]
Lipids	104.	Linoleic acid	280.45	[28]
	105.	Oleic acid	282.46	[28]
	106.	Palmitic acid	256.43	[28]
	107.	Stearic acid	284.48	[28]
	108.	Caffeic acid	180.16	[28]
Organic acids	109.	Malic acid	134.09	[28]
	110.	Benzoic acid	122.12	[28]
	111.	Citric acid	192.12	[25]
	112.	α-Linolenic acid	278.43	[29]
	113.	Dodecanoic acid	200.32	[28,29]
Vitamins	114.	Ascorbic acid	176.12	[2]
	115.	Thiamin chloride	300.81	[2]
	116.	Riboflavin	376.37	[2]
	117.	Pyridoxine	169.18	[2]
	118.	Nicotinic acid	123.11	[2]
	119.	D-Pantothenic acid	219.24	[27,68]
	120.	Folic acid	441.40	[27]
	121	Campesterol	400.68	[27,68]
Sterols	122.	beta-Sitosterol	414.71	[28]
	123.	Delta-7-Avenasterol	412.69	[28]
	124.	Phylloquinone	450.70	[28]
	125.	Eleutheroside A	576.85	[28]
	126.	α-Tocopherol	430.71	[25,70]
Other miscellaneous constituents	127.	L-Glutamic acid	147.13	[29]
	128.	L-Aspartic acid	133.10	[76]
	129.	L(+)-Arginine	174.20	[76]
	130.	Microcrystalline cellulose	324.28	[71]
	131.	Hemicellulose	148.11	[77]
	132.	Pectin	150.13	[2,77]
	133.	Amygdalin	457.43	[2]
	134.	Hydrogen cyanide	27.03	[78]
	135.	Benzaldehyde	106.12	[2]
	136.	Benzyl alcohol	108.14	[2]
	137.	2-Phenylethanol	122.17	[2]
	138.	Phenylacetaldehyde	120.15	[2]
	139.	3-Penten-2-one	84.12	[70]
	140.	1-Hexanol	102.18	[79]
	141.	trans-2-Hexen-1-ol	100.16	[75]
	142.	(E)-Anethole	148.20	[29]
	143.	Nonanal	142.24	[75]
	144.	Methyl arachidate	270.46	[28]
	145.	Methyl linoleate	294.48	[27]
	146.	Ethyl-2-[(3,4-dihydroxybenzoyloxy)-4,6-dihydroxyphenyl] acetate	348.31	[79]
	147.	Ethyl-2-methyl butanoate	130.18	[75]
	148.	Ethyl-3-methyl butanoate	130.18	[75]
	149.	Ethyl decanoate	200.32	[29]
	150.	Di-caffeoyl coumaroyl spermidine	615.70	[29]
	151.	β-Carotene	536. 88	[28,29]
	152.	Lycopene	536.85	[24]
	153.	Lutein	568.87	[24]
	154.	5,6-Epoxylutein	584.87	[24,75]
	155.	Neoxanthin	600.88	[24]
	156.	trans-Violaxanthin	600.87	[24]
	157.	cis-Violaxanthin	600.87	[24]
	158.	Fructose	180.16	[2]
	159.	Glucose	180.16	[2]
	160.	Sorbitol	182.17	[2]

Robust antioxidant activity. Structural characterization of the principal anthocyanidins identified cyanidin derivatives as the primary contributors to this antioxidant effect [28]. The concentrations of major bioactive compounds, including flavonols, flavanols, and anthocyanins, in different matrices (berry, juice, and pomace) are summarized in Table 2, along with the analytical methods used for their determination.

#### 4.1.1. Flavanones and Flavanonols

**Figure 1:**
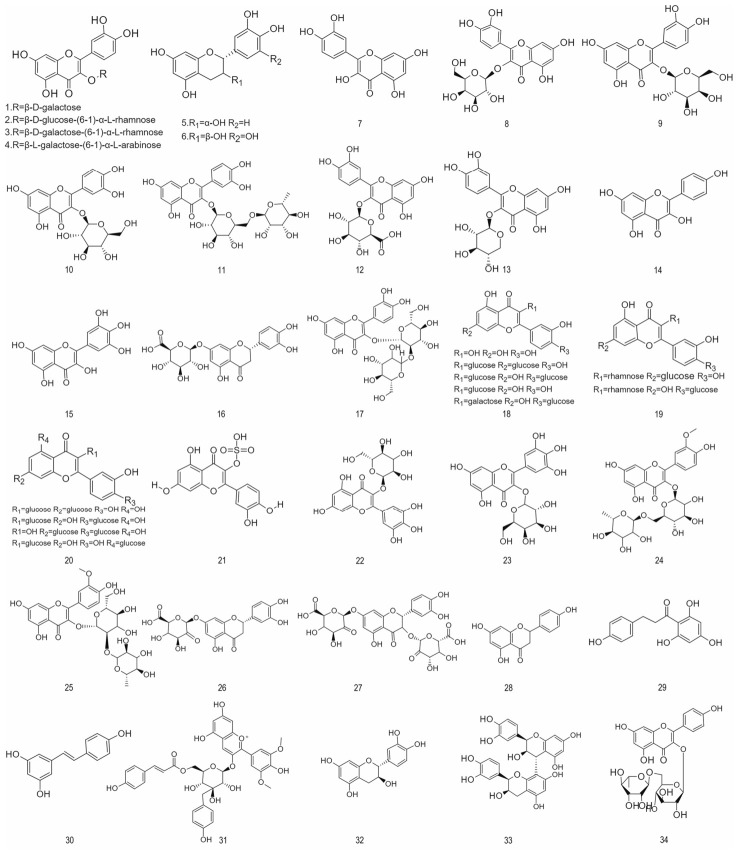
Major flavonoids in *A. melanocarpa* flavonols and flavanols. (Numbers correspond to the entries in Table 1).

Flavanones and flavanonols are two important classes of natural flavonoid compounds, found primarily in citrus fruits and various medicinal plants [80]. Typical examples of flavanones, such as naringin and hesperidin, possess significant antioxidant and anti-inflammatory activity, capable of scavenging free radicals and alleviating oxidative stress damage. Clinical studies have shown that they help improve microcirculation and reduce capillary fragility, playing a positive role in maintaining cardiovascular health. Furthermore, flavanones also help regulate lipid metabolism, contributing to a reduction in blood cholesterol levels [81]. Flavanones are one of the main subclasses of flavonoids and possess a range of beneficial effects, such as aiding blood glucose control, modulating lipid and renal function biomarkers, and regulating signaling pathways, thereby enhancing insulin sensitivity and glucose uptake and consequently helping to prevent diabetes and its associated complications. Consequently, flavanones are promising candidates with potential for the treatment of diabetes, although their efficacy still needs to be validated through human studies [82]. Figure 1 illustrates the chemical structures of flavanones and other flavonoids found in aronia.

#### 4.1.2. Anthocyanins

**Figure 2:**
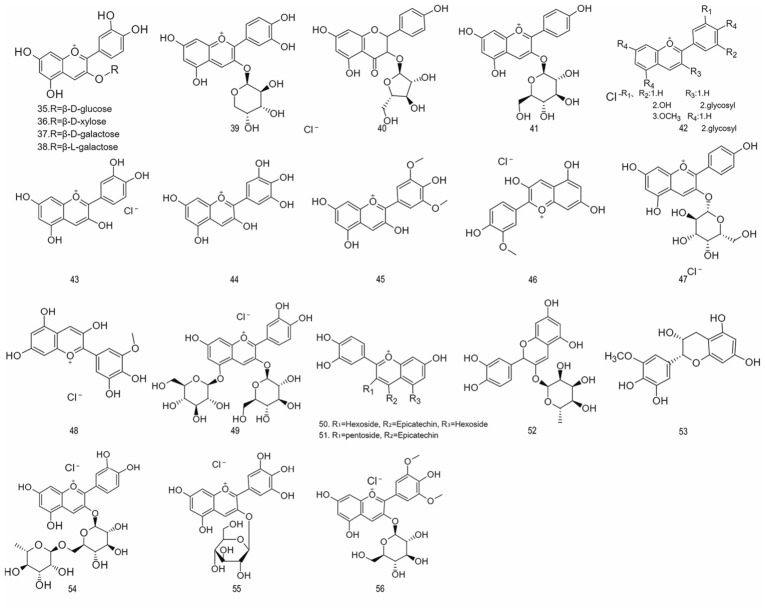
Major anthocyanidins and anthocyanins in *A. melanocarpa*. (Numbers correspond to the entries in Table 1).

Anthocyanins are among the most widely distributed water-soluble natural pigments in nature. They belong to the flavonoid class and impart vibrant red, purple, blue, and other colors to fruits, vegetables, and flowers. In recent years, driven by the growing demand for natural and healthy products among consumers, as well as on-going debates regarding the safety of synthetic pigments, anthocyanins have gained significant attention for their dual properties as both colorants and health-promoting agents. In the medical field, anthocyanins exhibit various biological activities, including antioxidant, anti-inflammatory, and antibacterial effects, as well as the ability to regulate gut flora. In the food industry, they serve both as an ideal source of natural coloring agents and play important roles in functional foods and smart active packaging [83]. The major anthocyanidins and anthocyanins identified in aronia are depicted in Figure 2.

#### 4.1.3. Phenolic Acids

**Figure 3:**
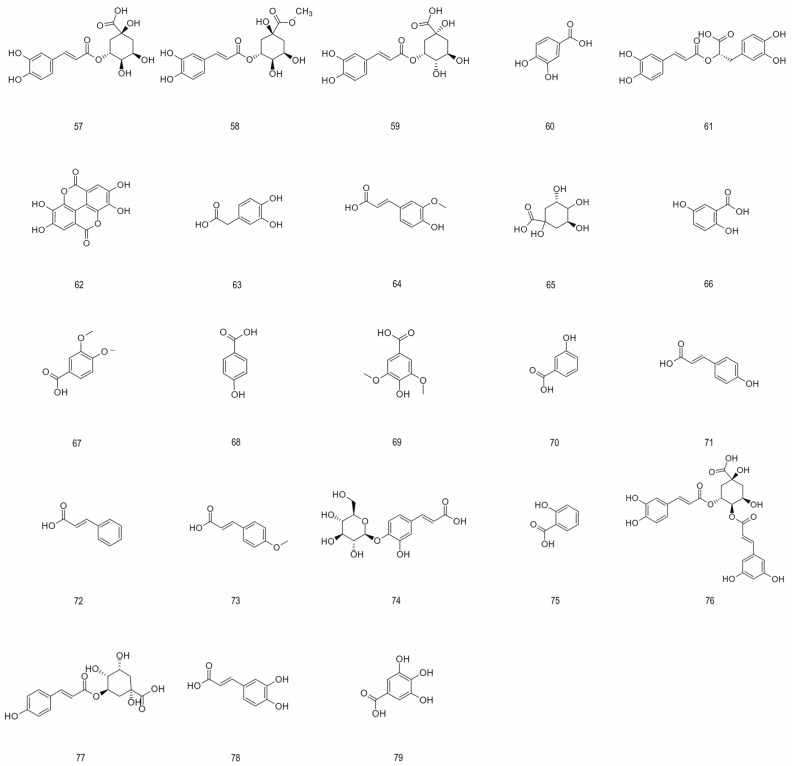
Structural types of phenolic acids in *A. melanocarpa*. (Numbers correspond to the entries in Table 1).

Phenolic acids represent a substantial and widely distributed class of phenolic compounds that exhibit significant biological activity. These activities include protecting the cardiovascular system, regulating the gut microbiota, improving glucose metabolism, and exerting a neuroprotective effect. They are primarily categorized into C6-C1 and C6-C3 skeletal structures. Phenolic acids constitute 7.5% of chokeberry polyphenols, with chlorogenic acid being the primary compound detected in the fruit. Research indicates that phenolic acid concentration in juice (808.9 mg per 100 g dry weight) exceeds that in pomace (373.6 mg per 100 g dry weight), demonstrating its favorable water solubility [25]. Figure 3 provides an overview of the structural types of phenolic acids present in aronia.

### 4.2. Terpenoid Compounds

**Figure 4:**
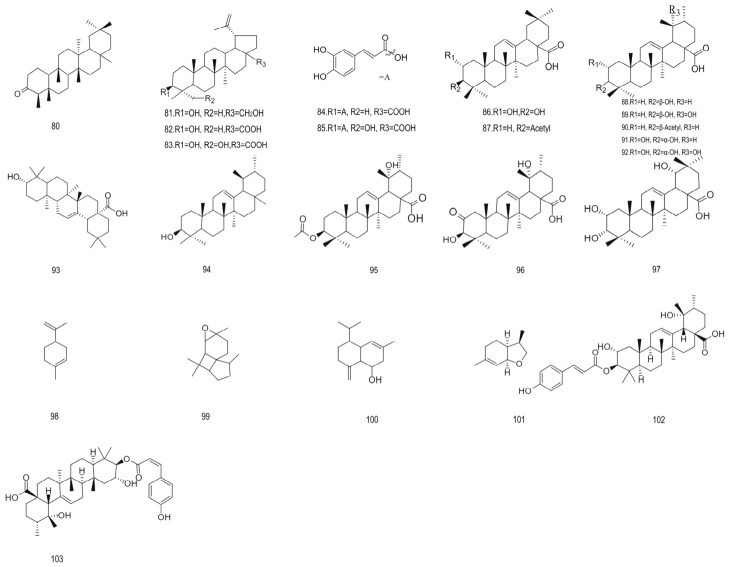
Representative terpenoids in *A. melanocarpa*. (Numbers correspond to the entries in Table 1).

Terpenoids are one of the most widely distributed and diversified natural products in nature. In the plant group, terpenoids play a central role in regulating morphological events during development [84,85]. Extraction of leaves and berry tissues with 95% ethanol revealed the presence of numerous triterpene derivatives (23.6–23.2% of the extract), including ittalene (17.2%), fenol (10.5%), and limonene (9.7%) [29,75]. The researchers also pointed out that these combinations of isopentane (C-5) secondary metabolites are heterogeneous and rich, exhibiting structural diversity that is unparalleled among natural substances. Empirical studies have shown that these compounds have a wide range of pharmacological effects: they have been confirmed to have antioxidant activity, inhibiting microbial proliferation, regulating inflammatory cascade response, reducing allergic reaction, inhibiting the proliferation of carcinogenic cell groups, limiting metastasis diffusion, interfering with angiogenesis and inducing it. Based on these accumulated data, terpenoids hold significant practical promise, with applications spanning food, dermatological, pharmaceutical, and therapeutic fields [86]. Figure 4 provides an overview of the structural types of terpenoids present in aronia.

### 4.3. Lipids and Organic Acid Compounds

Lipids are key metabolites synthesized in plants. They are central to biochemical and metabolic processes. Not only do lipids supply principal energy reserves and constitute foundational material for bilayer membrane architecture, but the interrelation of their anabolism is implicated as intrinsically linked with cycles involving organic acid matrices [87]. Analytical evidence specifies that oils expressed from Aronia present substantial proportions of phospholipids, sterols, and tocopherols; moreover, investigation into seed and residual fractions identifies a dominance of polyunsaturated fatty acids (nearly three-quarters of total fatty content), preeminently linoleic acid.

**Figure 5:**
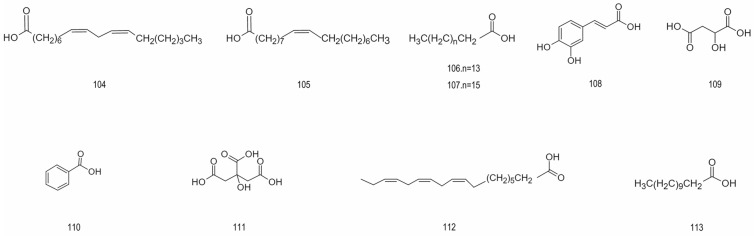
Key lipids and organic acids in *A. melanocarpa*. (Numbers correspond to the entries in Table 1).

Organic acids are also important metabolites synthesized in plants and play a central role in biochemical and metabolic processes. A variety of organic acids can be used as precursor substrates to regulate lipid biosynthesis through complementary reactions. The main organic acids in fresh wild aronia are L-malic acid and citric acid, and the contents of vanillic acid, oxalic acid and succinic acid are low [25]. From the perspective of dietary components, the coexistence of lipids and organic acids significantly affects the sensory characteristics such as taste and freshness of fruits [88]. Figure 5 provides an overview of the structural types of lipids and organic acids present in aronia.

### 4.4. Vitamins and Sterol Compounds

**Figure 6:**
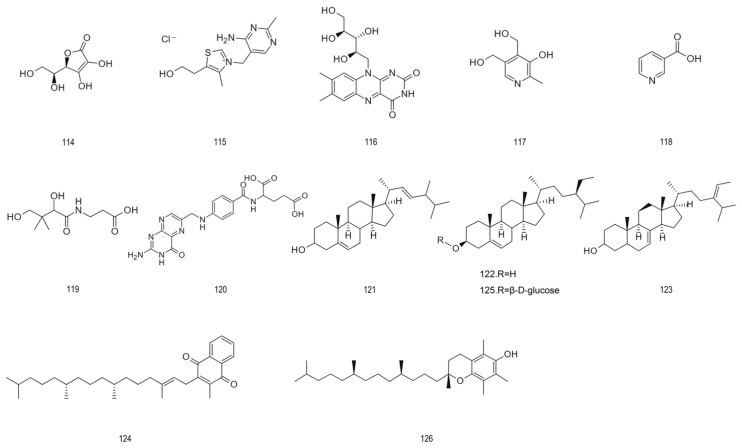
Vitamins and sterols in *A. melanocarpa*. (Numbers correspond to the entries in Table 1).

The evaluation of unprocessed juice samples showed that the content of vitamins B1, B2 and B6 in each liter of the sample, as well as the content of ascorbic acid, pantothenic acid and niacin, although there were fluctuations, always remained at a considerable micromolar level [25]. Vitamin C content in aronia fruit powder has been reported to be approximately 10 mg per gram [89]. Substances derived from aronia fruit seeds can provide beta-sitosterol and delta-7-avenasterol; fruit analysis mainly shows that beta-sitosterol and daucosterol exist together. Vitamins mainly act as enzyme cofactors to support physiological functions by promoting a variety of catalytic reactions [90]. Sterols play a structural role in cell membranes and serve as precursors for the synthesis of steroid hormones and vitamin D, contributing to the maintenance of homeostasis and immune regulation [91]. Figure 6 provides an overview of the structural types of vitaminic and steroidal compounds present in aronia.

### 4.5. Other Chemical Components

**Figure 7:**
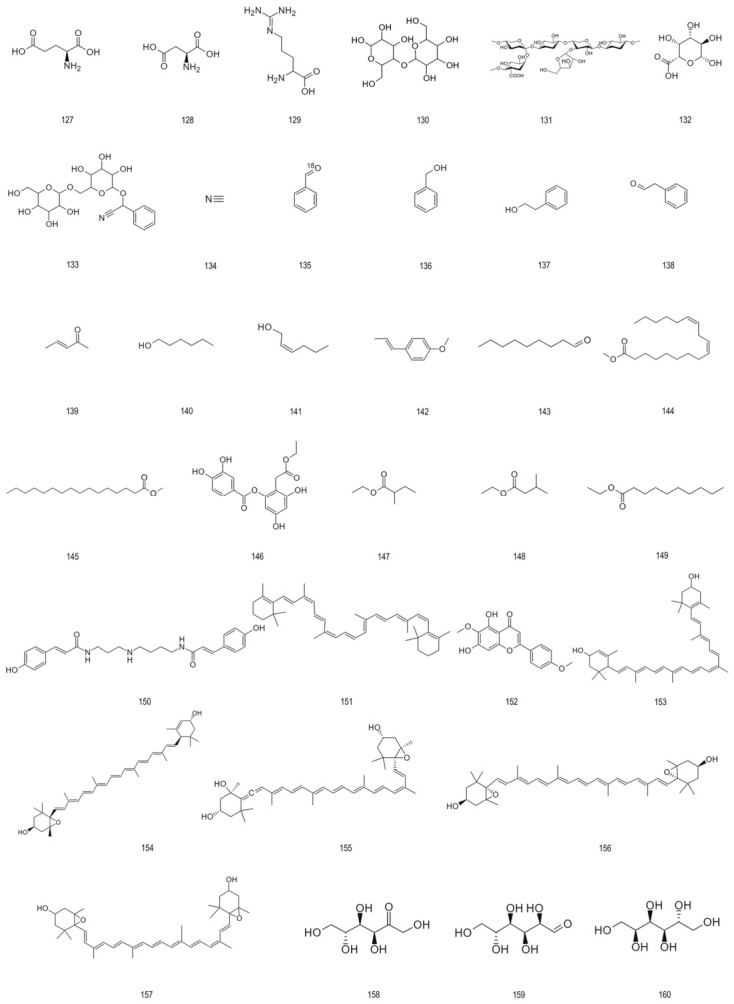
Other bioactive compounds in *A. melanocarpa*. (Numbers correspond to the entries in Table 1).

Constituent analyses taking into account a spectrum wider than the primary bio-actives have revealed the endogenous existence, in aronia, of amino-acid residues, fibrous polysaccharide elements, aromatic hydrocarbons, saccharidic polymers, and functionalized minor moieties. The quantitative determination assigns 0.049 mg/g ca-rotenoid to participants (predominantly lutein at two-thirds composition) within the fruits, whose species have been found to be utilized in visual health defense mechanisms as well as immunoregulatory procedures. Investigational focus directed to vola-tile elementality—inspired by Gas chromatography–mass spectrometry (GC-MS) signal quantification strategies—shows coverage of approximately one-fortieth of the total peak area by fatty acids, with alcohols achieving slightly less prominence; profiling uncovers thirty-six discrete volatiles categorically organized into twelve groups, collectively comprising over four-fifths of cumulative volatile fractionation. Although such trace or minority constituents rarely approximate the prevalence seen in canonical actives, interpretive syntheses highlight their ensemble contribution to overall biopharmacological effectiveness [92]. Figure 7 provides an overview of the structure.

## 5. Pharmacological Mechanisms

The beneficial health effects of aronia are mainly linked to its high concentrations of polyphenols, especially anthocyanins like cyanidin-3-galactoside. These compounds help regulate key signaling pathways in the body—such as Nrf2/KEAP1, NF-κB/MAPK, and PI3K/Akt—that control antioxidant defenses, inflammation, and cell survival. Numerous studies have shown that aronia extracts and their bioactive components exhibit a wide range of pharmacological effects, including antioxidant, anti-inflammatory, hepatoprotective, antitumor, neuroprotective, anti-fibrotic, and antidiabetic activities. The molecular mechanisms underlying these effects are summarized in Table 3, which presents the biological activities, experimental models, key targets, and signaling pathways reported for different aronia extracts and compounds. In the following sections, these mechanisms are discussed in more detail in the context of specific physiological and pathological conditions.

### 5.1. Antioxidant Effects

Oxidative stress is defined as the condition in which there is an overproduction of reactive oxygen species (ROS) that exceeds the capacity of endogenous antioxidants to neutralize them. It plays an essential role in cell senescence and in the development of multiple chronic pathological conditions, such as metabolic syndrome, neurodegener-ation and cardiovascular diseases [93,94]. Aronia is recognized as one of the richest di-etary sources of polyphenols among berries, and its antioxidant properties have been extensively studied [31].

**Figure 8:**
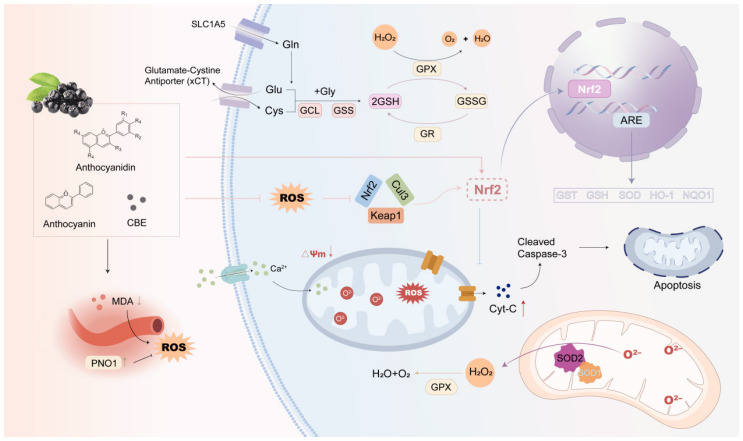
Antioxidant effects of *A. melanocarpa* with related molecular mechanisms. Aronia polyphenols activate the Nrf2 pathway to upregulate antioxidant enzymes while also directly scavenging free radicals and enhancing the glutathione system. (CBE, chokeberry extract; MDA, malondialdehyde; PNO1, partner of NOB1; ROS, reactive oxygen species; SLC1A5, solute carrier family 1 member 5; Gln, glutamine; Glu, glutamate; Cys, cysteine; Gly, glycine; GCL, glutamate-cysteine ligase; GSS, glutathione synthetase; GSH, glutathione; GPX, glutathione peroxidase; GR, glutathione reductase; GSSG, oxidized glutathione; Nrf2, nuclear factor erythroid 2-related factor 2; Keap1, Kelch-like ECH-associated protein 1; Cul3, cullin 3; ARE, antioxidant response element; GST, glutathione S-transferase; SOD, superoxide dismutase; HO-1, heme oxygenase-1; NQO1, NAD(P)H quinone dehydrogenase 1; ΔΨm, mitochondrial membrane potential; Cyt-C, cytochrome c; Caspase-3, cysteine-aspartic acid protease 3; SOD1, superoxide dismutase 1; SOD2, superoxide dismutase 2).

The antioxidant activity of aronia is primarily based on activating the Nrf2/Keap1 pathway. Its bioactives cause the release of Nrf2 from its cytoplasmic inhibitor Keap1, facilitating Nrf2 nuclear translocation. then associates with antioxidant response elements (ARE) to drive the transcription of gene products for critical phase II detoxifying and antioxidant enzymes such as heme oxygenase-1 (HO-1), NAD(P)H quinone dehydrogenase 1 (NQO1), superoxide dismutase (SOD), glutathione peroxidase (GPx) and glutathione S-transferase (GST) [32,95]. These enzymes work together for the elimination of ROS such as superoxide anion (O_2_•−) and hydrogen peroxide (H2O2). Both in vitro and in vivo studies have supported these effects, showing reduced ROS levels and increased antioxidant enzyme activity following aronia treatment [31,90,95,96]. In addition to Nrf2 activation, aronia polyphenols remove free radicals and complexes with pro-oxidant metals, as well as increase the cellular glutathione (GSH) pool through up-regulation of the xCT transporter. In addition, they display protective effects at the mitochondrial level through stabilization of mitochondrial membrane potential (ΔΨm) thus preventing the mitochondrial permeability transition pore opening and consequent apoptotic cascade [95]. Figure 8 illustrates the coordinated antioxidant actions of aronia polyphenols. They activate the Nrf2 pathway to boost antioxidant enzymes like HO-1 and SOD, while also directly scavenging free radicals and enhancing the glutathione system to maintain cellular redox balance.

**Table 3:** Biological properties of extracts or compounds from *A. melanocarpa* and their potential mechanisms of action. (In the “Key Result” column, ↑ indicates an in-crease in the parameter, while ↓ indicates a decrease).

Extract/Compound	Key Result	Mechanism/Pathway	Activity	Model (In Vivo)	Model (In Vitro)	References
ABE	↑cell cycle arrest; ↑DNA damage	p53/Chk1	Anticancer		SW480, HCT116, PDO cells, colorectal cancer model	[38]
AC	↓edema; ↓TNF-α/IL-1β/IL-6; ↓ICAM-1	ROS-MAPK-NF-κB	Anti-inflammatory	Mouse, TPA-induced ear edema model		[97]
	↓edema; ↓TNF-α/IL-1β/IL-6; ↓ICAM-1	ROS-MAPK-NF-κB	Anti-inflammatory		HaCaT cells, skin inflammation model	[97]
AJ	↑SOD/CAT; ↑p-Akt; improves learning/memory	PI3K/Akt	Neuroprotective	Mouse, D-galactose-induced aging model	PC12 cells, H_2_O_2_-induced oxidative injury model	[90]
AJ (F5)	↓Aβ; ↓BACE1 activity; improves cognition	BACE1 inhibition	Neuroprotective	Mouse, 5XFAD Alzheimer’s disease model		[98]
AMA	↓α-SMA; ↓collagen; ↓TNF-α, ↓IL-1β; ↓HSC activation	TGF-β/Smad; inflammatory pathways	Anti-fibrotic	Mouse, CCl_4_-induced hepatic fibrosis model		[99,100]
	↓ROS; ↓iNOS; ↓SOCS3; improves insulin resistance	IKKβ/NF-κB; JAK2/Stat3/Stat5B	Hepatoprotective	Mouse, HFD/STZ-induced T2DM model		[41]
	↓AST/ALT; ↓COX-2; ↓IL-6; ↑GSH-PX; ↑Bcl-2	α7nAChR/PI3K/Akt; Nrf2/HO-1	Hepatoprotective	Mouse, alcohol-induced liver disease (ALD) model		[35,101,102]
	↑SOD/GSH-PX; ↓MDA; ↑NE/DA/5-HT; ↓COX-2/IL-1β	Oxidative stress; neurotransmitter modulation	Anti-cognitive decline	Mouse, aging-accelerated model		[90]
	↓proliferation; ↓colorectal injury; ↓inflammation; ↓GLS/SLC1A5	mTORC1; glutamine metabolism	Anticancer	Mouse, AOM/DSS-induced colitis-associated CRC model	Caco-2 cells, colorectal cancer model	[103]
	↓proliferation; ↑E-cadherin	Wnt/β-catenin; mitochondrial apoptosis	Anticancer		Caco-2 cells, colorectal cancer model	[39]
	↑Nrf2; ↑HO-1/SOD/GSH; ↓ROS; ↓Cyto c	Nrf2/Keap1/ARE	Antioxidant		LPS-stimulated macrophages, inflammation model	[96]
AMAE	↓blood glucose; ↓ROS; ↓inflammation; ↑glucose	IKKβ/NF-κB; JAK2/Stat3/5B	Anti-diabetic	Mouse, HFD/STZ-induced T2DM model		[41]
	↑glycogen; ↑glycolysis; ↓gluconeogenesis; ↓lipids; ↓ROS; ↓inflammation	PI3K/Akt; GLUT2; PPARγ; AMPK	Anti-diabetic	Rat, HFD/STZ-induced T2DM model		[104]
	↑glucose uptake; ↑insulin sensitivity; ↑glycogen synthesis; ↓SOCS3	SOCS3-dependent insulin signaling	Anti-diabetic		IR HepG2, IR C2C12 cells, insulin resistance model	[105]
AMPs	↓AST/ALT; ↓ROS; ↓Bax; ↑Bcl-2; ↑ZO-1; ↓TNF-α/IL-6/IL-1β	TLR4/MyD88; STAT3	Hepatoprotective	Rat, LPS-induced liver injury model		[106]
	↑Nrf2; ↑SOD/CAT/GSH-PX; ↓TNF-α/IL-6/IL-1β; ↓MDA/4-HNE; ↓FPN	Nrf2-Keap1; TLR4-MyD88	Hepatoprotective	Rat, LPS-induced liver injury model		[107]
	↑BDNF modulates gut microbiota	Gut–brain axis	Anti-depressive	Mouse, corticosterone-induced depression model		[108]
Anthocyanins	↓ROS; ↑Nrf2; ↑Bcl-2; ↓Bax/Casp-3	Nrf2; mitochondrial apoptosis	Neuroprotective		SH-SY5Y cells, Aβ-induced neurotoxicity model	[109]
BCPs, AMPs	↑ZO-1/Occludin/Claudin-1; ↓TNF-α/IL-6/IL-1β; modulates microbiota	TLR4/MyD88/NF-κB/STAT3	Hepatoprotective/Anti-inflammatory	Rat, LPS-induced liver injury model; Mouse, DSS-induced colitis mode		[107,110]
BCPs	↓dyslipidemia; ↓steatosis; modulates gut microbiota; alters lipid metabolites	Gut microbiota; glycerophospholipid metabolism	Anti-obesity	Rat, HFD-induced obesity model		[111]
C3G	↑E-cadherin; ↓α-SMA; ↓EMT	NRF2/HO-1; TGF-β/mTOR	Anti-fibrotic	Mouse, silica-induced pulmonary fibrosis model		[99,112]
	↓TNF-α/IL-1β/IL-6; ↓Cyto c; promotes mitophagy	NF-κB/MAPK; Pink1/Parkin	Anti-inflammatory	Mouse, PM10-induced acute lung injury model		[113]
C3G, BCPs	↓TNF-α/IL-6/IL-1β; improves metabolic dysregulation	AMPK; STAT3/NF-κB	Anti-obesity/Anti-inflammatory	Rat, HFD-induced obesity model		[114]
CBPs	↓obesity; ↓dyslipidemia; modulates gut microbiota; alters bile acids; ↑thermogenesis; FMT improves dyslipidemia	FXR/TGR-5; thermogenesis	Anti-obesity	Rat, HFD-induced obesity model		[111]
CBE, ARN, AME	↓NO/PGE2; ↓TNF-α/IL-6/IL-1β; ↓iNOS/COX-2	NF-κB; MAPK; Nrf2	Anti-inflammatory		LPS-stimulated macrophages, inflammation model	[115,116]
CBE	↓cholesterol uptake; ↑cholesterol efflux; ↑LDL uptake; ↓lipogenesis	Cholesterol metabolism; SIRT1/3/5	Anti-obesity		Caco-2 cells, cholesterol metabolism model	[117,118,119]
Extracts	↓ICAM-1/VCAM-1; ↓ROS	NF-κB	Anti-inflammatory		TNF-α-stimulated endothelial cells, vascular inflammation model	[120,121]

C3G, cyanidin-3-galactoside; AMA, *Aronia melanocarpa* anthocyanins; AMPs, *Aronia melanocarpa* polyphenols; AJ, aronia juice; AC, aronia concentrate; CBE, chokeberry extract; BCPs, black chokeberry polyphenols; CBPs, chokeberry poly-phenols; AMAE, *Aronia melanocarpa* anthocyanin extracts; ABE, aronia berry extract; α-SMA, alpha-smooth muscle actin; EMT, epithelial–mesenchymal transition; HSC, hepatic stellate cell; T2DM, type 2 diabetes mellitus; ALD, alcoholic liver disease; LPS, lipopolysaccharide; CCl4, carbon tetrachloride; AST, aspartate aminotransferase; ALT, alanine aminotransferase; COX-2, cyclooxygenase-2; IL, interleukin; TNF-α, tumor necrosis factor alpha; GSH-PX, glutathione peroxidase; Bcl-2, B-cell lymphoma 2; Bax, Bcl-2-associated X protein; ROS, reactive oxygen species; iNOS, inducible nitric oxide synthase; SOCS3, suppressor of cytokine signaling 3; IKKβ, IκB kinase β; NF-κB, nu-clear factor kappa B; JAK2, Janus kinase 2; STAT3, signal transducer and activator of transcription 3; Nrf2, nuclear factor erythroid 2-related factor 2; Keap1, Kelch-like ECH-associated protein 1; HO-1, heme oxygenase-1; SOD, superoxide dismutase; CAT, catalase; MDA, malondialdehyde; 4-HNE, 4-hydroxynonenal; FPN, ferroportin; TLR4, toll-like receptor 4; MyD88, myeloid differentiation primary response 88; ZO-1, zonula occludens-1; Occludin, tight junction protein; Claudin-1, tight junction protein; BDNF, brain-derived neurotrophic factor; ICAM-1, intercellular adhesion molecule 1; VCAM-1, vascular cell adhesion molecule 1; MAPK, mitogen-activated protein kinase; PI3K, phosphatidylinositol 3-kinase; Akt, protein kinase B; AMPK, AMP-activated protein kinase; GLUT2, glucose transporter type 2; GLUT4, glucose transporter type 4; PPARγ, peroxisome proliferator-activated receptor gamma; FXR, farnesoid X receptor; TGR5, Takeda G protein-coupled receptor 5; FMT, fecal microbiota transplantation; AOM, azoxymethane; DSS, dextran sodium sulfate; CRC, colorectal cancer; GLS, glutaminase; SLC1A5, solute carrier family 1 member 5; SLC7A11, solute carrier family 7 member 11; mTORC1, mechanistic target of rapamycin complex 1; Chk1, checkpoint kinase 1; p53, tumor protein p53; IR, insulin resistance; HFD, high-fat diet; PGE2, prostaglandin E2; NO, nitric oxide; 4EBP, eIF4E-binding protein; ATR, ataxia telangiectasia and Rad3-related protein; β-TrCP, beta-transducin repeat containing protein; CK1α, casein kinase 1 alpha; eIF4E, eukaryotic translation initiation factor 4E; p70S6K, p70 S6 kinase; RPS6, ribosomal protein S6; ABE, aronia berry extract.

### 5.2. Anti-Inflammatory Effects

Chronic inflammation, which involves a complicated pathological process, has been related to the pathogenesis of many diseases, including the metabolic syndrome, cardiovascular disease, neurodegeneration and dermatological disorders. Evidence suggests that the extract of aronia and its polyphenol constituents, mainly anthocyanins like cyanidin-3-O-galactoside (C3G) and cyanidin-3-O-glucoside, exhibit substantial anti-inflammatory activity in a range of in vitro and in vivo models [32,97,116,120,122,123,124,125,126].

The anti-inflammatory effects of aronia are primarily mediated through inhibition of the NF-κB signaling pathway. Aronia concentrates (AC) and extracts efficiently inhibit the tumor necrosis factor-alpha (TNF-α)-induced IκB kinase (IKK) activation and IκBα degradation, as well as its subsequent phosphorylation, nuclear translocation, and DNA-binding activity of the NF-κB p65 subunit in HaCaT and HAECs [97,123]. In addition, aronia bioactive fractions attenuate MAPK and STAT3 activation, further contributing to the suppression of inflammatory signaling [97,114,127].

The antioxidant capacity of aronia polyphenols plays a synergistic role. By scavenging intracellular ROS and activating the Nrf2 pathway, they mitigate ROS-sensitive activation of NF-κB and MAPK [32,97,122,123]. These mechanisms are supported by in vivo evidence. Topical administration of AC attenuated TPA-induced mouse ear edema [97], while oral administration of C3G or anthocyanin-rich extracts reduced high-fat diet-induced inflammation and suppressed NF-κB phosphorylation in rodents [114,116,122,125].

Emerging evidence also indicates that aronia polyphenols modulate the gut microbiota, increasing beneficial genera such as Akkermansia, enhancing intestinal barrier integrity, and promoting the production of anti-inflammatory short-chain fatty acids (SCFAs) [120,122,125,128]. This modulation of the gut-liver axis and gut–brain axis is then followed by a dampening of systemic endotoxemia and Toll-like receptor 4 (TLR4)/NF-κB-driven inflammation in distal organs [122,125].

**Figure 9:**
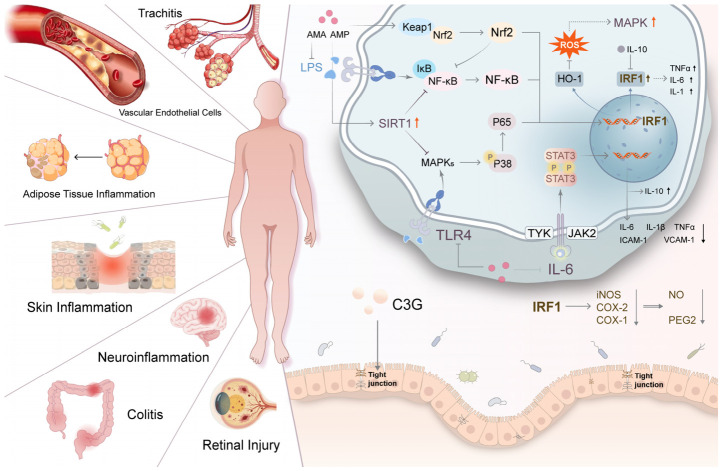
Anti-inflammatory effects of *A. melanocarpa* with related molecular mechanisms. Aronia compounds suppress NF-κB and MAPK signaling, reducing inflammatory cytokines, while also repairing the gut barrier and alleviating systemic inflammation. (AMA, *Aronia melanocarpa* anthocyanins; AMP, *Aronia melanocarpa* polyphenols; LPS, lipopolysaccharide; Keap1, Kelch-like ECH-associated protein 1; Nrf2, nuclear factor erythroid 2-related factor 2; NF-κB, nuclear factor kap-pa-light-chain-enhancer of activated B cells; IκB, inhibitor of nuclear factor kappa-B; SIRT1, sirtuin 1; MAPK, mitogen-activated protein kinase; P38, p38 mitogen-activated protein kinase; P65, RELA proto-oncogene, NF-kB subunit (p65); TLR4, toll-like receptor 4; TYK, tyrosine kinase; JAK2, Janus kinase 2; STAT3, signal transducer and activator of transcription 3; IRF1, interferon regulatory factor 1; ROS, reactive oxygen species; HO-1, heme oxygenase-1; IL-1, interleukin-1; IL-6, interleukin-6; IL-10, inter-leukin-10; IL-1β, Interleukin-1β; TNFα, tumor necrosis factor alpha; ICAM-1, inter-cellular adhesion molecule 1; VCAM-1, vascular cell adhesion molecule 1; C3G, cya-nidin-3-glucoside; iNOS, inducible nitric oxide synthase; COX-2, cyclooxygenase-2; COX-1, cyclooxygenase-1; NO, nitric oxide; PEG2, prostaglandin E2).

Together, these results suggest that the anti-inflammatory action of aronia is based on an integrated approach with both direct down-regulation of pro-inflammatory transcription factors and kinases, antioxidants in cells, and beneficial effects on gut microorganisms. The anti-inflammatory effects of aronia are summarized in Figure 9, which shows how these compounds suppress NF-κB and MAPK signaling to reduce inflammatory cytokines, while also helping to repair the gut barrier and reduce systemic inflammation.

### 5.3. Hepatoprotective Effects

Liver damage is caused by various reasons like excessive intake of alcohol and chemicals (for example, CCl4 and lipopolysaccharide (LPS)) and metabolism disorders with complicated pathophysiology including oxidative stress, inflammatory response, and apoptosis. Evidence indicates that aronia extract and especially the *Aronia melanocarpa* polyphenol-rich fractions (AMPs) as well as *Aronia melanocarpa* anthocyanin fractions (AMAs) showed considerable protection in different models of liver damage.

The hepatoprotective effects of aronia are primarily attributed to its antioxidant and anti-inflammatory activities. One major mechanism involves activation of the Nrf2 pathway. Aronia bioactives promote Nrf2 nuclear translocation, leading to up-regulation of downstream antioxidant enzymes such as HO-1, SOD, catalase (CAT), and glutathione peroxidase (GSH-Px). This action reduces ROS and malondialdehyde (MDA) levels while increasing GSH levels in the liver, thereby alleviating oxidative stress [35,114,129,130,131].

Meanwhile, aronia extracts block the activation of NF-κB and MAPK signaling pathways, leading to reduced release of the pro-inflammatory cytokine TNF-α, IL-6 or IL-1β from damaged liver cells [35,107]. In addition, they regulate PI3K/Akt and STAT3 signaling pathways. The activation of PI3K/Akt induces cell survival and sup-presses apoptosis, while STAT3 regulation could either suppress inflammation or in-duce apoptosis [35,132,133].

Interestingly, treatment with aronia was found to specifically reduce ferroptosis, a new type of iron-mediated programmed cell death involved in hepatic damage. Quer-cetin is a natural product that inhibits ferroptosis via the IL-6/STAT3 pathway, and it is found in large quantities in aronia. In addition, quercetin has demonstrated anti-ferroptotic effects in models of acute liver injury and restoration of glutathione peroxidase 4 (GPX4) expression [134].

Both in vitro and in vivo studies support these protective effects. In vitro studies have demonstrated that aronia extracts reduce oxidative stress and inflammation in liver cell models [107,132]. In vivo studies have shown that aronia extracts improve liver histology, reduce serum levels of liver damage markers (ALT and AST), improve lipid profiles, and reduce hepatocyte apoptosis in models of alcoholic liver disease (ALD), LPS-induced injury, and CCl4 intoxication [35,107,132,135,136,137].

In addition to direct effects on liver cells, aronia polyphenols exert a beneficial effect through the gut-liver axis. They modulate the intestinal microbiota by increasing intestinal barrier function through up-regulation of tight junction proteins (ZO-1, Occludin, claudin-1) and decreasing systemic endotoxemia (e.g., serum LPS levels), which in turn alleviates TLR4/MyD88/NF-κB signaling in the liver [107].

**Figure 10:**
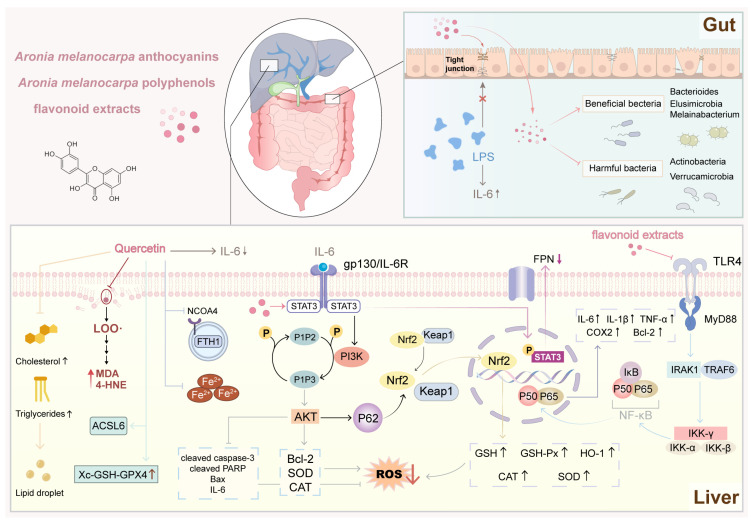
Hepatoprotective effects of *A. melanocarpa* with related molecular mechanisms. Aronia compounds activate Nrf2, suppress NF-κB, and inhibit ferropto-sis to protect the liver, while also restoring gut microbiota balance to further support liver function. (LPS, lipopolysaccharide; IL-6, interleukin-6; IL-1β, interleukin-1 beta; TNF-α, tumor necrosis factor alpha; gp130/IL-6R, glycoprotein 130/interleukin-6 re-ceptor; FPN, ferroportin; TLR4, toll-like receptor 4; MyD88, myeloid differentiation primary response 88; LOO·, lipid peroxyl radical; MDA, malondialdehyde; 4-HNE, 4-hydroxynonenal; ACSL6, acyl-CoA synthetase long chain family member 6; Xc-GSH-GPX4, cystine/glutamate antiporter-glutathione-glutathione peroxidase 4; NCOA4, nuclear receptor coactivator 4; FTH1, ferritin heavy chain 1; cleaved caspase-3, cleaved cysteine-aspartic acid protease 3; cleaved PARP, cleaved poly (ADP-ribose) polymerase; Bax, BCL2-associated X protein; STAT3, signal transducer and activator of transcription 3; PIP2, phosphatidylinositol 4,5-bisphosphate; PIP3, phosphatidyl-inositol 3,4,5-trisphosphate; PI3K, phosphoinositide 3-kinase; AKT, protein kinase B; Bcl-2, B-cell lymphoma 2; SOD, superoxide dismutase; CAT, catalase; ROS, reactive oxygen species; P50, NF-κB subunit p50; P62, sequestosome 1; P65, NF-κB subunit p65; IκB, inhibitor of nuclear factor kappa-B; Nrf2, nuclear factor erythroid 2-related factor 2; Keap1, Kelch-like ECH-associated protein 1; NF-κB, nuclear factor kap-pa-light-chain-enhancer of activated B cells; IRAK1, interleukin-1 receptor-associated kinase 1; TRAF6, TNF receptor-associated factor 6; IKK-γ, inhibitor of nuclear factor kappa-B kinase subunit gamma; IKK-α, inhibitor of nuclear factor kappa-B kinase subunit alpha; IKK-β, inhibitor of nuclear factor kappa-B kinase subunit beta; COX2, cyclooxygenase 2; GSH, glutathione; GSH-Px, glutathione peroxidase; HO-1, heme oxygenase-1).

Overall, the hepatoprotection of aronia is achieved by coordinating regulation of the Nrf2/HO-1, PI3K/Akt and NF-κB/STAT3 pathway, in combination with suppression of ferroptosis and enhancement of the intestinal barrier, making it an attractive multifunctional agent in the prevention and adjunct therapy of chemical and metabolism-induced liver injury. Figure 10 integrates the hepatoprotective mechanisms of aronia. By activating Nrf2, suppressing NF-κB, and inhibiting ferroptosis, these compounds protect the liver from damage—and they also help restore a healthy balance of gut microbes, which further supports liver function.

### 5.4. Antitumor Effects

Aronia and its active compounds, especially AMA and other anthocyanins as well as flavonoids such as quercetin, have shown promising antiproliferative/pro-apoptotic effects in a variety of cancer cell lines, including colorectal (Caco-2) or breast and endometrial carcinoma cell lines through several different modes of action that culminate in the inhibition of oncogenic signaling pathways including induction of programmed cell death and disruption of cancer cell metabolism [37,38,39,103,138].

The first and main target is the wingless-related integration site (Wnt)/β-catenin pathway which plays a crucial role during CRC development. In vitro studies in Ca-co-2 cells have shown that AMA has no impact on the overall level of β-catenin, but it strongly interferes with their co-activating ability, facilitating the degradation of free cytoplasmic ß-catenin, probably through its inhibition of phosphorylating Glycogen Synthase Kinase 3 Beta (GSK3β), thereby promoting β-catenin proteasomal degradation, resulting in a profound downregulation of β-catenin/T-cell factor 4 (TCF4) transcriptional activity and subsequent reduction in expression of its downstream onco-genic targets including c-Myc, Cyclin D1 and Survivin [39].

A secondary mechanism is the induction of mitochondrial apoptosis. AMA in-creases the Bax/Bcl-2 ratio, promotes cytochrome c release, and activates caspase-3, leading to intrinsic apoptosis in cancer cells [39]. In addition, in vitro studies have shown that aronia berry extract (ABE) induces cell cycle arrest and enhances DNA damage repair, possibly through the regulation of Chk1 and p53 pathways [38].

Other contributing mechanisms include the promotion of ferroptosis and the disruption of cancer metabolism. In vitro studies in breast and endometrial cancer cells have demonstrated that quercetin induces ferroptosis in breast and endometrial cancer cells by promoting TFEB nuclear translocation, which leads to lysosomal degradation of ferritin, increased intracellular labile iron, and accumulation of lipid per-oxides [37,138]. Furthermore, aronia bioactives suppress glutamine metabolism by in-hibiting the expression of the glutamine transporter SLC1A5 and the metabolic enzyme GLS, reducing α-ketoglutarate production and ATP synthesis. This metabolic stress is accompanied by suppression of the mTORC1 pathway, ultimately inhibiting protein synthesis and tumor growth [103].

In addition to direct anti-proliferative effects, aronia constituents reduce inflam-mation at the tumor site. They decrease neutrophil influx (MPO) and down-regulate pro-inflammatory cytokines (TNF-α, IL-6, IL-17, IFN-γ) and enzymes (COX-2) in colon cancer models, thereby dampening cancer-promoting inflammation [103].

**Figure 11:**
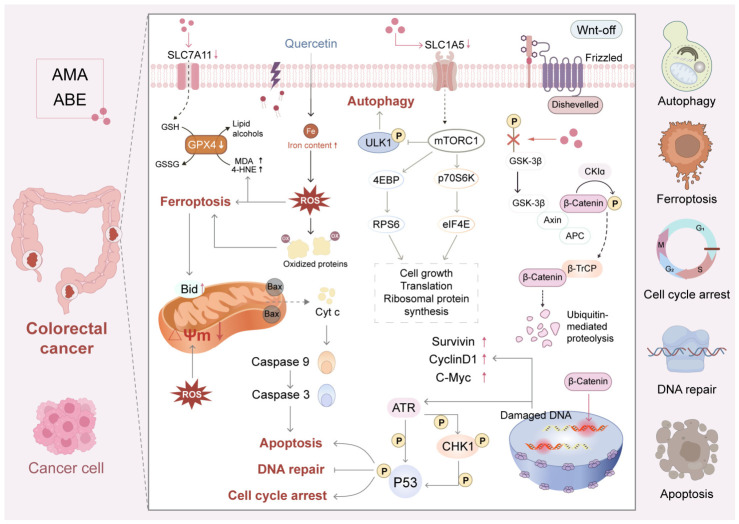
Anti-cancer effects of *A. melanocarpa* with related molecular mechanisms. Aronia compounds inhibit cancer cell growth via suppressing Wnt/β-catenin, inducing mitochondrial apoptosis, disrupting cancer cell metabolism, and activating p53 tumor suppressor. (AMA, *Aronia melanocarpa* anthocyanins; ABE, *Aronia melanocarpa* extract; SLC7A11, solute carrier family 7 member 11; SLC1A5, solute carrier family 1 member 5; GPX4, glutathione peroxidase 4; GSH, glutathione; GSSG, oxidized glutathione; MDA, malondialdehyde; 4-HNE, 4-hydroxynonenal; ROS, reactive oxygen species; ΔΨm, mitochondrial membrane potential; Bid, BH3 interacting-domain death agonist; Bax, BCL2-associated X protein; Cyt c, cytochrome c; Caspase 9, cysteine-aspartic acid protease 9; Caspase 3, cysteine-aspartic acid protease 3; ULK1, unc-51 like autophagy activating kinase 1; mTORC1, mechanistic target of rapamycin complex 1; 4EBP, eukaryotic translation initiation factor 4E-binding protein; p70S6K, p70 ribosomal protein S6 kinase; RPS6, ribosomal protein S6; eIF4E, eukaryotic translation initiation factor 4E; GSK-3β, glycogen synthase kinase 3 beta; CK1α, casein kinase 1 alpha; APC, adenomatous polyposis coli; β-TrCP, beta-transducin repeat containing E3 ubiquitin protein ligase; ATR, ataxia telangiectasia and Rad3-related kinase; CHK1, checkpoint kinase 1; P53, tumor protein p53).

In summary, aronia exerts its anticancer effects by targeting several vulnerabilities of tumor cells: inhibition of the oncogenic Wnt/β-catenin pathway, induction of mitochondrial apoptosis and ferroptosis, inhibition of pro-tumorigenic inflammation, and disruption of the critical metabolism/anabolism pathways required for rapid proliferation. The antitumor effects of aronia are depicted in Figure 11. These compounds work through multiple pathways—including suppression of the Wnt/β-catenin path-way, induction of mitochondrial apoptosis, disruption of cancer cell metabolism, and activation of the p53 tumor suppressor—to inhibit cancer cell growth.

### 5.5. Neuroprotective Effects

Cognitive loss and neurodegeneration in aging, Alzheimer’s disease (AD), epilepsy, and depression have multifactorial pathogenesis that includes oxidative stress, neuro-inflammation, mitochondrial dysfunction, aberrant protein aggregation and dysregulated cell death pathways. In vitro and in vivo studies suggest that aronia ex-tracts, especially the anthocyanin-rich extract (cyanidin-3-O-galactoside, cya-nidin-3-O-arabinoside) juice show promise as neuroprotectants in response to such injuries by multiple means, including a central role in inducing strong antioxidant responses at the level of individual cells, namely the transcriptional activator Nrf2 and its repressor Keap1 [90,98,108,129,130,131,139].

Aronia treatments up-regulate Nrf2 expression and nuclear translocation resulting in the up-regulation of downstream antioxidants including HO-1, NQO1, SOD, CAT, and GSH, which can reduce the level of ROS and MDA in the brain [101,140]. This anti-oxidative barrier is further supported by up-regulation of survival-signaling pathways such as PI3K/Akt. In vitro studies in PC12 cells and in vivo studies in aged mice have shown that activation of PI3K/Akt signaling by aronia juice improves neuronal viability, promotes cell cycle progression, and inhibits apoptosis [90].

In in vitro and in vivo models of AD pathogenesis, anthocyanins from aronia have been shown to protect cells against the neurotoxic effects of amyloid-beta (Aβ) through reducing the damage caused to mitochondria via maintaining their mem-brane potential and ATP content, regulating intracellular calcium homeostasis, and suppressing the mitochondrial apoptotic cascade. This is accomplished via up-regulation of the anti-apoptotic B-cell lymphoma 2 (Bcl-2) and down-regulation of the pro-apoptotic Bcl-2-associated X protein (Bax) with a consequent reduction in the cytochrome c release and caspase-3/9 activation [140,141]. In particular, in vivo studies in AD model mice have shown that specific anthocyanins are able to block the activity of asparagine endopeptidase (AEP)—another enzyme responsible for cutting up amyloid precursor protein (APP) into Aβ peptides and which acts via the δ-secretase pathway, thus decreasing Aβ deposit in the brain of AD model mice [130].

Furthermore, there are some recent findings that indicate that aronia could play a role in other types of regulated cell death apart from apoptosis. For instance, in vivo studies in a kainic acid-induced epilepsy model have shown that one of its main polyphenolic compounds, quercetin, exerts an anti-ferroptotic effect on an animal model of epilepsy induced with kainic acid. Quercetin can protect against seizure-induced neuronal death and memory deficits through the activation of the Sirtuin 1 (SIRT1)/Nrf2 pathway, which then up-regulates the cystine/glutamate antiporter (So-lute carrier family 1 member 5 (SLC7A11)) and GPX4, two of the main defenses against ferroptosis [129].

Apart from the direct neuroprotective effect, aronia polyphenols also have a beneficial impact on the body through the gut–brain axis. For example, in vivo studies in depressed mice have shown that administration of an extract of aronia polyphenols to depressed mice was able to positively modulate the gut microbiota by increasing its diversity and modifying the Firmicutes/Bacteroidetes ratio as well as significantly in-creasing the levels of brain-derived neurotrophic factor (BDNF), connecting the gut with brain protection and emotional states [108].

These mechanistic results are complemented by behavioral data demonstrating that aronia juice (or anthocyanins) enhances spatial learning, memory, and learning and exploring ability of aged and AD model animals as well as anticonvulsant activity with improvement of cognition in epilepsy model animals [90,98,129,130].

**Figure 12:**
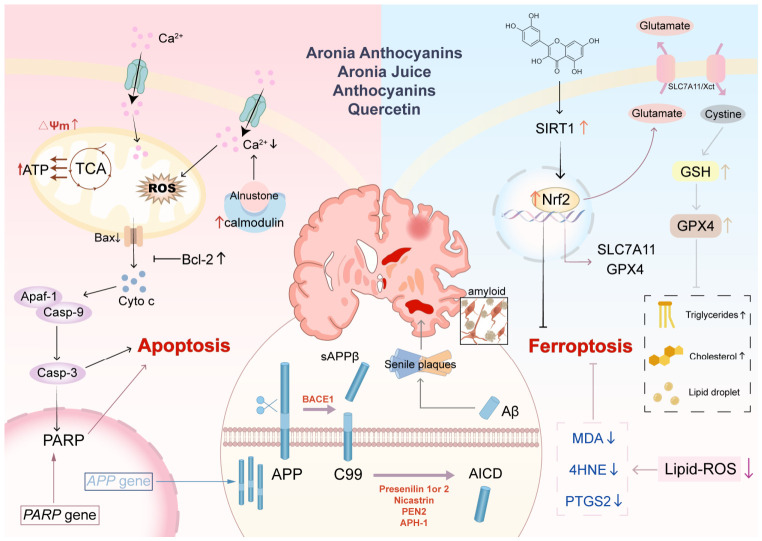
Neuroprotective effects of *A. melanocarpa* with related molecular mechanisms. Aronia compounds activate Nrf2, inhibit amyloid-β plaque formation, and suppress apoptotic and ferroptotic cell death pathways, thereby protecting neurons and supporting cognitive function. (ΔΨm, mitochondrial membrane potential; ATP, adenosine triphosphate; TCA, tricarboxylic acid cycle; ROS, reactive oxygen species; Bax, Bcl-2-associated X protein; Bcl-2, B-cell lymphoma 2; Cyto c, cytochrome c; Apaf-1, apoptotic peptidase activating factor 1; PARP, poly(ADP-ribose) polymerase; APP, amyloid precursor protein; sAPPβ, soluble amyloid precursor protein β; BACE1, β-site APP-cleaving enzyme 1; C99, C-terminal fragment 99; Aβ, amyloid beta; AICD, APP intracellular domain; SIRT1, sirtuin 1; Nrf2, nuclear factor erythroid 2-related factor 2; SLC7A11, solute carrier family 7 member 11; Xct, system Xc−; GSH, glutathi-one; GPX4, glutathione peroxidase 4; MDA, malondialdehyde; 4HNE, 4-hydroxynonenal; PTGS2, prostaglandin-endoperoxide synthase 2; Lipid-ROS, lipid reactive oxygen species; APH-1, anterior pharynx defective 1 homolog; PEN2, prese-nilin enhancer 2).

Overall, the neuroprotective/cognitive benefits of aronia may be achieved by a combined approach including increasing the levels of antioxidants with Nrf2 stimulation, neuronal survival through the activation of the PI3K/Akt pathway, inhibition of Aβ production/toxicity, suppression of apoptotic and ferroptotic cell death, and the promotion of good gut–brain dialog. Figure 12 summarizes the neuroprotective effects of aronia. By activating Nrf2, inhibiting amyloid-β plaque buildup, and suppressing both apoptotic and ferroptotic cell death pathways, these compounds help protect neurons and support cognitive function.

### 5.6. Anti-Fibrotic Effects

**Figure 13:**
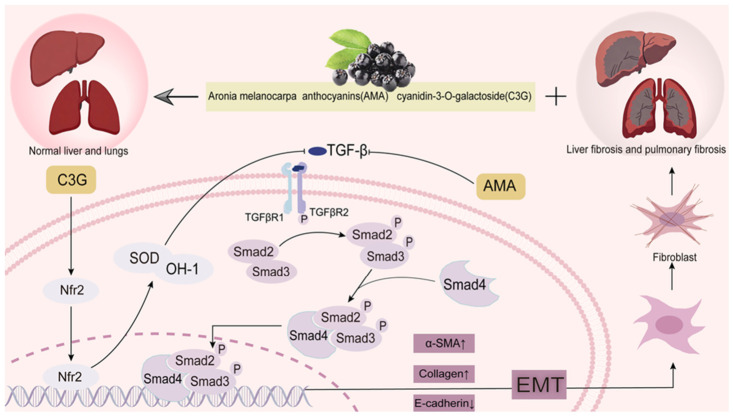
Anti-fibrotic effects of *A. melanocarpa* with related molecular mechanisms. Aronia compounds suppress TGF-β/Smad signaling to reduce collagen deposition and activate Nrf2/HO-1 to counteract oxidative damage. (AMA, *Aronia melanocarpa* anthocyanins; C3G, cyanidin-3-O-galactoside; TGF-β, transforming growth factor-beta; TGFβR1, transforming growth factor-beta receptor 1; TGFβR2, transforming growth factor-beta receptor 2; SOD, superoxide dismutase; OH-1, heme oxygenase-1; Nrf2, nuclear factor erythroid 2-related factor 2; Smad2, mothers against decapentaple-gic homolog 2; Smad3, mothers against decapentaplegic homolog 3; Smad4, mothers against decapentaplegic homolog 4; α-SMA, alpha-smooth muscle actin; EMT, epithelial–mesenchymal transition).

Aronia’s antifibrotic effect, primarily attributed to its anthocyanins, includes a concerted modulatory action on two main routes in models of pulmonary and hepatic fibrosis [112,142,143], which is through a dual control over the antioxidant Nrf2/HO-1 axis and the profibrotic transforming growth factor-beta (TGF-ß)/Smad signaling network [100,112]. In vivo studies in silica-induced pulmonary fibrosis models and CCl4-induced hepatic fibrosis models have shown that this dual effect counteracts oxidative tissue injury as well as blocks critical fibrogenic processes, including inhibition of fibroblast and hepatic stellate cell (HSC) activation and proliferation, repressing epithelial-to-mesenchymal transition (EMT), and decreasing overproduction and deposition of extracellular matrix (ECM) molecules, particularly collagen [112,142]. The concurrent down-regulation of pro-inflammatory cytokines, such as TNF-α, further facilitates the establishment of an anti-fibrotic microenvironment. The anti-fibrotic effects of aronia are illustrated in Figure 13. These compounds work by suppressing TGF-β/Smad signaling to reduce collagen buildup while activating Nrf2/HO-1 to protect against oxidative damage.

### 5.7. Anti-Diabetic and Anti-Obesity Effects

T2DM and obesity are interconnected metabolic diseases defined as insulin resistance, low-grade inflammation, oxidative stress and dysregulated lipid/glucose metabolism. Aronia extracts especially the anthocyanin-rich (AMAE) and polyphenol-rich fraction show promise for treating such diseases by acting on several targets simultaneously [41,104,105,111,117,118,119,144,145,146].

A major action is related to the improvement of insulin sensitivity and glucose homeostasis. In vitro studies in hepatic (HepG2) and muscle (C2C12) cells have shown that aronia extracts induce the activation of the insulin signaling pathway in hepatic and muscle cells by increasing the phosphorylation of insulin receptor substrate (IRS), PI3K, Akt and glycogen synthase kinase-3b (GSK-3b), thus promoting glycogen syn-thesis and inhibiting gluconeogenesis. Simultaneously, they upregulate expression and membrane translocation of glucose transporters (glucose transporter type 2 (GLUT2) and glucose transporter type 4 (GLUT4)) facilitating cellular glucose uptake [104,105,146]. Crucially, these effects are partly mediated by downregulation of Suppressor of Cytokine Signaling 3 (SOCS3) which is a negative regulator of insulin signaling, which is overexpressed in the state of IR [41,105]. In vivo studies in T2DM rodent models have further confirmed that aronia extracts rectify hepatic glucose metabolism by enhancing the activity of glycolytic enzymes (hexokinase, glucokinase and pyruvate kinase) while suppressing gluconeogenic enzymes (phosphoenolpyruvate carboxykinase and glucose-6-phosphatase) [104,146].

In parallel, aronia exerts hypolipidemic effects. Lowers the serum and liver contents of triglyceride (TG), total cholesterol (TC), low-density lipoprotein cholesterol (LDL-C), and free fatty acids (FFAs) but increases high-density lipoprotein cholesterol (HDL-C) [124,125,126]. In vitro studies in adipocytes and in vivo studies in obese rodent models have shown that this occurs through the suppression of important adipogenic transcription factors (Peroxisome proliferator-activated receptor gamma (PPARγ), CCAAT/enhancer-binding protein alpha (C/EBP), and sterol regulatory element-binding protein 1c (SREBP1c)) and lipogenic enzymes (e.g., fatty acid synthase) in adipocytes and the liver, thus blocking de novo lipogenesis and adipocyte differentiation [117].

Underlying these metabolic improvements are robust antioxidant and anti-inflammatory actions. In vitro and in vivo studies have shown that aronia bioactive compounds scavenge ROS and GSH and decrease MDA, thus reducing oxidative stress [41,104,118]. They also inhibit the activation of pro-inflammatory signaling cas-cades (IKK/NF-κB, JAK2/STAT3), which leads to a reduction in cytokine production, including TNF-α, IL-6 and IL-1β in metabolic tissues [41,104].

An emerging and significant mechanism is the modulation of the gut-liver axis. In vivo studies in diet-induced obese rats have demonstrated that aronia polyphenols beneficially reshape gut microbiota composition—increasing the abundance of Akkermansia, Bacteroides, and Prevotella while decreasing the Firmicutes/Bacteroidetes ratio. This remodeling improves intestinal barrier function, decreases systemic endo-toxemia and modulates host BAs metabolism. These changes in BA profile (e.g., in-creased chenodeoxycholic acid) trigger intestinal Farnesoid X Receptor (FXR) and Takeda G Protein-Coupled Receptor 5 (TGR5) signaling, which in turn regulates he-patic gluconeogenesis and lipogenesis as well as the energy expenditure of brown adipose tissue [111].

Additionally, in vivo studies in T2DM models have shown that quercetin protects from pancreatic ß-cell dysfunction through blocking a new pathologic mechanism: ferroptosis. Quercetin mitigates iron overload, restores GPX4 activity, and reduces lipid peroxidation in β-cells, thereby preserving insulin secretory capacity [145]. Additionally, in vitro studies have indicated that aronia polyphenols inhibit the formation of advanced glycation end products (AGEs), which are implicated in diabetic complications, by scavenging free radicals and protecting protein structure [118].

**Figure 14:**
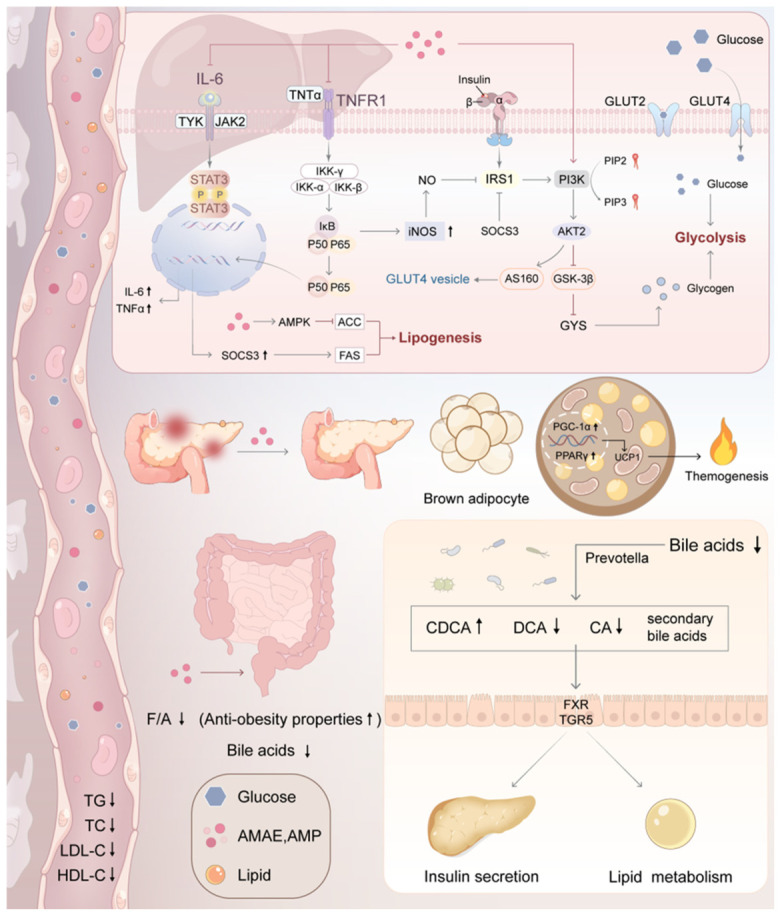
Anti-diabetic and anti-obesity effects of *A. melanocarpa* with related molecular mechanisms. Aronia compounds improve insulin sensitivity, regulate blood glucose and lipids, and promote a healthier gut microbiota, collectively contributing to better metabolic health. (AMAE, *Aronia melanocarpa* anthocyanin extract; AMP, *Aronia melanocarpa* polyphenols; IL-6, interleukin-6; TNFα, tumor necrosis factor-alpha; TNFR1, tumor necrosis factor receptor 1; TNT, Troponin T; TYK, tyrosine kinase; JAK2, janus kinase 2; STAT3, signal transducer and activator of transcription 3; IKK, IκB kinase; IκB, inhibitor of nuclear factor kappa B; P50, NF-κB subunit p50; P65, NF-κB subunit p65; AMPK, adenosine monophosphate-activated protein kinase; ACC, acetyl-CoA carboxylase; FAS, fatty acid synthase; SOCS3, suppressor of cytokine signaling 3; IRS1, insulin receptor substrate 1; NO, nitric oxide; tNOS, total nitric oxide synthase; PI3K, phosphoinositide 3-kinase; PIP2, phosphatidylinositol 4,5-bisphosphate; PIP3, phosphatidylinositol 3,4,5-trisphosphate; AKT2, protein kinase B 2; AS160, AKT substrate of 160 kDa; GSK-3β, glycogen synthase kinase 3 beta; GYS, glycogen synthase; GLUT2, glucose transporter 2; GLUT4, glucose transporter 4; PGC-1α, peroxisome proliferator-activated receptor gamma coactivator 1-alpha; PPARγ, peroxisome proliferator-activated receptor gamma; UCP1, uncoupling protein 1; CDCA, chenodeoxy-cholic acid; DCA, deoxycholic acid; CA, cholic acid; FXR, farnesoid X receptor; TGR5, takeda G-protein coupled receptor 5; TG, triglyceride; TC, total cholesterol; LDL-C, low-density lipoprotein cholesterol; HDL-C, high-density lipoprotein cholesterol; F/A, Firmicutes/Bacteroidetes ratio.).

Taken together, the antidiabetic and anti-obesity effects of aronia are based on an overall approach to improve insulin signaling, optimize glucose and lipid metabolism, quench oxidative stress and inflammation, beneficially modulate the gut microbiome and bile acid axis, and protect the pancreatic ß-cell against ferroptosis. Figure 14 pro-vides an integrated view of aronia’s metabolic benefits. These compounds improve insulin sensitivity, help regulate blood sugar and fats, and support a healthier balance of gut bacteria—all of which contribute to better metabolic health.

## 6. Food Application

The health benefits of aronia, which we discussed in Section 5—especially its ability to fight oxidation and reduce inflammation—help explain why it is effective in functional foods. Aronia shows great promise in the functional food industry due to its richness in nutrients, particularly polyphenols, anthocyanins, flavonoids, and phenolic acids, as well as dietary fiber, sorbitol, and vitamin C [141]. However, using aronia in real-world food products presents several challenges. The red color from anthocyanins can fade during processing and storage. The raw berries are very astringent, and heat treatment may reduce their health benefits [20,141]. To mitigate its natural astringent taste in raw form, aronia is often processed and is utilized in various derivative food products such as juice, yogurt, jam, syrup and tea, to bring out its basic nutritional value [9,10,11,12,13,14]. In addition to the basic nutritional value, it can also be used in other food fields due to its remarkable antioxidant properties, such as sports nutrition products, natural food antioxidant additives and natural food pigments for food preservation and food coloring [12,15,16,18,19,20,21,22,23].

### 6.1. Conventional Food Products

While the natural astringency of raw berries makes them unpalatable for direct consumption, they can be transformed through primary processing into a variety of consumer food products. This includes juice, beverages, yogurt, jam, syrup, and tea-based items, which significantly enhances their palatability and overall food value. For manufacturers, keeping the color stable over time and preserving the anthocyanin content during storage are key concerns [20,21].

#### 6.1.1. Juice

The most common form of aronia food is juice. This juice can be consumed as a standalone beverage or as a supplement to other fruit juices, enhancing the flavor and nutritional properties of the juice. Research has shown that aronia juice can be combined with lemon juice to create a beverage, which increases the retention rate of anthocyanins in the juice product, thereby enhancing its antioxidant activity [9]. At the same time, the fruit powder of aronia can also be added to the juices of other fruits to improve the taste and increase the nutritional value of the juice [14]. For example, adding the powder of fruit to apple juice can increase the vitamin C, total phenols, flavonoids and anthocyanins content of the juice, thereby enhancing its antioxidant capacity. When making juice on an industrial scale, factors like temperature, acidity, and processing time significantly influence anthocyanins’ stability [21,141]. Juice production targets high yield and anthocyanin content. Aronia contains much pectin. This pectin makes the pulp highly viscous. Pressing the juice becomes challenging. Using pectinase with ultrasonic assistance at 35 °C resulted in a juice yield of 41.33%, compared to a pulp yield of only 17.80%, and reduced anthocyanin loss to 40.02% [140].

#### 6.1.2. Yogurt

The probiotic food industry also has its place in the food application sector. Yogurt is one type of probiotic product. The beneficial synergy between aronia bioactives and microbial cultures, incorporating aronia juice, pulp or pomace into fermentation yields a premium yogurt with enhanced quality and potential probiotic benefits [11]. It is known that the high polyphenol content and antioxidant capacity in aronia juice have a prebiotic effect, which can enhance the antioxidant ability and survival ability of probiotics. Researchers added aronia juice to fermented milk with probiotics. The milk became significantly more acidic. Total phenolic content stayed higher in this yogurt. Yogurt without aronia juice had lower phenolic levels. The yogurt with fermented aronia juice rich in probiotics has a high content of probiotics and lactic acid, adding value to the yogurt product [13]. Moreover, the addition of pulp and juice will increase the richness of the yogurt’s texture, make the color more appealing to consumers, and maintain the protein content in the yogurt, thus strengthening the commercial value of yogurt [10]. In yogurt production, when and how much aronia is added (before or after fermentation) can affect both the growth of beneficial bacteria and the final product’s stability [11,13].

### 6.2. Sports Nutrition Supplements

Aronia can also be used to make sports supplements due to its rich polyphenol content. Phenol supplements can improve various aspects of an athlete’s health and ultimately enhance their performance [18]. Due to the presence of polyphenols in aronia which have antioxidant properties, aronia supplements can be made to act as antioxidants, reducing the levels of markers indicative of lipid peroxidation induced by exercise, altering the redox balance in the athlete’s body, and modifying antioxidant enzymes [15]. Studies have shown that aronia juice can be used as an antioxidant supplement for football players, helping them achieve an oxidative stress balance in their bodies [17]. Aronia sports supplements can help alleviate exercise-induced oxidative stress. This effect appears to be mediated through inhibiting platelet function and iron metabolism, subsequently leading to attenuated platelet activation and counteracting anemia. For marathon and half-marathon athletes, the restoration of platelet function and iron metabolism levels is critical. This restoration not only helps mitigate the risk of cardiac arrest but also alleviates the heightened platelet reactivity caused by long-distance running [12]. Beyond supporting competitive athletes, aronia supplements can also benefit individuals engaging in unaccustomed, prolonged exercise. Furthermore, aronia supplements can offer protection against adverse effects caused by long-term training regimens like those for triathlons by influencing the changes in serum heparin levels [16]. For supplement manufacturers, making sure the extract dose is consistent and that the active ingredients stay stable in the final product are important practical issues [15,18].

### 6.3. Food Additives

The food industry values aronia not only for its nutritional profile but also for its abundance of powerful antioxidant phytochemicals [141]. Extracted bioactive components can be formulated into natural food additives that can replace synthetic preservatives like nitrite. This approach offers a non-toxic method for food quality preservation and shelf-life extension, underscoring its significant market potential [19].

#### 6.3.1. Food Coloring

Aronia is rich in anthocyanins and is a good source of natural pigments [21]. Juice processing generates a significant amount of pomace as a by-product which was previously regarded as waste material. This by-product is increasingly recognized as a valuable source of natural pigments. Supercritical CO_2_ extraction with ethanol as a co-solvent was performed at 40 MPa and 90 °C. About 2.7 g of lipophilic and phenolic compounds were produced per 100 g of dried fruit residue [147]. The pigments extracted from pomace are safe and have the natural color property of food due to high amounts of anthocyanins. After pressing, the anthocyanins are highly retained in the fruit residue, and the color stability is strong. Therefore, the fruit residue, as a by-product of juice processing, can be efficiently utilized for the extraction of natural pigments in the food industry, enhancing the commercial value of food. In addition, the fruit powder obtained by grinding the fruit residue can also be used as a natural coloring material [22]. For large-scale pigment extraction, factors such as solvent type, temperature, and extraction time need to be optimized to maximize color yield and stability [21,22].

#### 6.3.2. Natural Food Antioxidant Additives

The anthocyanins in aronia have antibacterial and antioxidant properties and can be used to produce natural food antioxidant additives, which can be employed to extend the shelf life of food. Extracts from aronia’s leaf and fruit pulp are abundant in antibacterial substances. These compounds suppress the proliferation of spoilage and pathogenic bacteria in meat, thereby extending the shelf life of meat products. Notably, due to its safety profile, aronia can be used as a food preservative to replace chemical preservatives such as nitrites, providing a non-toxic option for food safety [19]. The utility of these extracts extends to innovative preservation formats. The polyvinyl alcohol (CP) film contained 8% AME. Its tensile strength reached 26.79 MPa, the highest value. Elongation at break was 66.38%. This film also exhibited strong antioxidant and antibacterial activities [148]. Research has shown that the extracted anthocyanins boost the efficacy of preservation films for pork. Meat spoilage is assessed by pH value. Spoilage occurs once the pH exceeds 6.7. Experimental results at 4 °C showed the following. AMA was added to the S/P/A/T film group. This slowed the pH rise in pork. Shelf life was extended from 6 days to nearly 9 days [23]. Coating from the extract of aronia flowers effectively preserves fresh fruit slices by preventing oxidative degradation [20]. For practical applications, it is important to evaluate the stability of these active compounds in different food types and under various storage conditions [19,23].

## 7. Clinical Application

Compared to well-studied berries like blueberries, the clinical evidence for aronia is still limited [149,150,151,152]. Blueberries have been tested in many human trials looking at heart health, brain function, and metabolism. For aronia, substantially fewer studies are available; most involve small sample sizes, and there is considerable variability across studies in both the type of product used (e.g., whole fruit, juice, or extract) and the administered doses [141,143,144,153,154]. Despite these limitations, the available human evidence still provides meaningful insights into the potential health benefits of aronia.

### 7.1. Heart Health

Studies have found that aronia may benefit heart health. One randomized controlled trial in healthy men showed that taking aronia extract (providing 116 mg of polyphenols per day) or whole fruit powder (providing 12 mg of polyphenols per day) for 12 weeks helped lower blood pressure and improve blood vessel function [155,156].

### 7.2. Brain Function

For brain function, a randomized controlled trial in older adults who were overweight or obese found that taking anthocyanin-rich aronia extract (40 mg of anthocyanins per day) for 6 weeks improved executive function and working memory [153]. Another study in healthy middle-aged people showed that long-term use of aronia extract (90 mg or 150 mg per day) for 24 weeks improved psychomotor speed [156].

### 7.3. Exercise Recovery and Antioxidant Defense

Aronia extract has also been shown to help athletes recover from exercise. It strengthens the body’s glutathione antioxidant system, reduces oxidative stress caused by intense exercise, increases total antioxidant capacity, and lowers inflammatory markers [157,158]. One study in young football players used 6 g of freeze-dried aronia extract per day for 90 days [158].

### 7.4. Blood Sugar and Metabolism

For blood sugar control, aronia does not seem to have a strong effect on fasting blood glucose levels. However, it has been shown to improve insulin sensitivity and lower blood sugar levels after meals, which may be especially helpful for people with prediabetes [154,159]. A study in prediabetic adults used a mixture containing aronia at a dose of 4 g twice daily for 12 weeks [159].

### 7.5. Other Possible Benefits

Animal studies suggest that aronia may protect the liver and kidneys from damage caused by environmental toxins like cadmium [160,161]. One small human study also found that a supplement containing aronia may improve near vision and reduce dry eye symptoms in people with presbyopia (age-related farsightedness) [162]. In addition, taking aronia has been linked to better gut health, with an increase in beneficial bacteria that may help improve blood vessel function [155].

### 7.6. Current Limitations

It is important to keep in mind that the current evidence has several limitations. Most studies are small (usually fewer than 100 people), short (6 to 12 weeks), and use different forms of aronia (juice, extract, or powder) with different amounts of active compounds. Notably, one study in T2DM patients did not report the exact dose of aronia used, only stating that the snack bar contained 37% aronia. This makes it hard to compare results across studies or to know exactly what dose works best. Larger, longer studies with standardized extracts are needed to confirm these early findings.

## 8. Conclusions and Perspectives

Existing evidence suggests that aronia is a rich source of polyphenolic compounds, which possess multiple bioactivities, such as antioxidant, anti-inflammatory, and metabolic regulatory properties. Although existing evidence supports its potential clinical value, large-scale, long-term studies are still needed to clarify the optimal dosage, underlying pharmacological mechanisms, and specific benefits across diverse populations. Moreover, there are still technical challenges concerning the stability, bioavailability, and processing of these bioactive compounds. Future work should focus on demonstrating the chemical transformations of key phenolics during food processing and storage, developing effective delivery strategies (e.g., encapsulation, nanoemulsions) to improve their bioavailability in complex food matrices, and systematically evaluating how formulation and processing influence the preservation and functionality of aronia bioactives in products. Such research will strengthen the scientific foundation for ingredient development based on aronia and support the creation of standardized, efficacious functional foods, contributing to human health.

## Figures and Tables

**Table 2:** Content of major bioactive compounds in *A. melanocarpa* berries, juice, and pomace, with corresponding analytical methods. (DES (deep eutectic solvent) is an ex-traction method used prior to HPLC analysis; it is not an analytical technique per se. Extracts prepared with DES were subsequently analyzed by HPLC, LC-MS, or SSNMR as indicated).

Name	Molecular Weight	Content (mg/100 g Dry Weight)			Analytical Method	References
Cyanidin-3-O-glucoside	468.84	0.3–42	28	79	HPLC; DES; SSNMR; LC-MS	[25,26,27]
Cyanidin-3-O-xyloside	386.36	53	34	105	HPLC; DES; SSNMR	[25]
Cyanidin-3-O-galactoside	466.40	19–1282	787	1120	HPLC-MS; DES; SSNMR; LC-MS	[25,26,27,28]
Quercetin-3-O-rutinoside	610.52	15	28	14	HPLC; LC-MSn	[25]
(−)-Epicatechin	290.27	15	13	11	HPLC; SSNMR	[25]
Quercetin	302.24	12–44			HPLC-DA; LC-MS	[69]
Quercetin-3-O-galactoside	464.38	37	50	47	HPLC-DAD; HPLC	[25,27]
Quercetin-3-O-glucoside	478.36	22	31	27	HPLC; LC-MSn	[25,68]
Quercetin-3-O-xyloside	434.35			0.5	HPLC	[25]

## Data Availability

No new data were created or analyzed in this study. Data sharing is not applicable to this article.
